# FDA Sodium Reduction Targets and the Food Industry: Are There Incentives to Reformulate? Microsimulation Cost‐Effectiveness Analysis

**DOI:** 10.1111/1468-0009.12402

**Published:** 2019-07-22

**Authors:** BRENDAN COLLINS, CHRIS KYPRIDEMOS, JONATHAN PEARSON‐STUTTARD, YUE HUANG, PIOTR BANDOSZ, PARKE WILDE, ROGAN KERSH, SIMON CAPEWELL, DARIUSH MOZAFFARIAN, LAURIE P. WHITSEL, RENATA MICHA, MARTIN O'FLAHERTY, on behalf of the Food‐PRICE Investigators

**Affiliations:** ^1^ University of Liverpool; ^2^ School of Public Health Imperial College London; ^3^ Friedman School of Nutrition Science and Policy Tufts University; ^4^ Medical University of Gdansk; ^5^ Wake Forest University; ^6^ American Heart Association

**Keywords:** cardiovascular disease, cost‐effectiveness analysis, food industry, health policy, sodium reduction

## Abstract

**Policy Points**

- The World Health Organization has recommended sodium reduction as a “best buy” to prevent cardiovascular disease (CVD). Despite this, Congress has temporarily blocked the US Food and Drug Administration (FDA) from implementing voluntary industry targets for sodium reduction in processed foods, the implementation of which could cost the industry around $16 billion over 10 years.
- We modeled the health and economic impact of meeting the two‐year and ten‐year FDA targets, from the perspective of people working in the food system itself, over 20 years, from 2017 to 2036.
- Benefits of implementing the FDA voluntary sodium targets extend to food companies and food system workers, and the value of CVD‐related health gains and cost savings are together greater than the government and industry costs of reformulation.

**Context:**

The US Food and Drug Administration (FDA) set draft voluntary targets to reduce sodium levels in processed foods. We aimed to determine cost effectiveness of meeting these draft sodium targets, from the perspective of US food system workers.

**Methods:**

We employed a microsimulation cost‐effectiveness analysis using the US IMPACT Food Policy model with two scenarios: (1) short term, achieving two‐year FDA reformulation targets only, and (2) long term, achieving 10‐year FDA reformulation targets.

We modeled four close‐to‐reality populations: food system “ever” workers; food system “current” workers in 2017; and subsets of processed food “ever” and “current” workers. Outcomes included cardiovascular disease cases prevented and postponed as well as incremental cost‐effectiveness ratio per quality‐adjusted life year (QALY) gained from 2017 to 2036.

**Findings:**

Among food system ever workers, achieving long‐term sodium reduction targets could produce 20‐year health gains of approximately 180,000 QALYs (95% uncertainty interval [UI]: 150,000 to 209,000) and health cost savings of approximately $5.2 billion (95% UI: $3.5 billion to $8.3 billion), with an incremental cost‐effectiveness ratio (ICER) of $62,000 (95% UI: $1,000 to $171,000) per QALY gained. For the subset of processed food industry workers, health gains would be approximately 32,000 QALYs (95% UI: 27,000 to 37,000); cost savings, $1.0 billion (95% UI: $0.7bn to $1.6bn); and ICER, $486,000 (95% UI: $148,000 to $1,094,000) per QALY gained. Because many health benefits may occur in individuals older than 65 or the uninsured, these health savings would be shared among individuals, industry, and government.

**Conclusions:**

The benefits of implementing the FDA voluntary sodium targets extend to food companies and food system workers, with the value of health gains and health care cost savings outweighing the costs of reformulation, although not for the processed food industry.

Poor diet is a major driver of ill health in the United States, with excess sodium being one of the key aspects.^1^ Average sodium intake in the United States is high (3,400 mg/day) and has been linked to an estimated 66,508 annual cardiometabolic deaths including from stroke and coronary heart disease (CHD).^1^ About 75% of this intake comes from processed and commercially produced foods,^2^ meaning that most sodium is under the control of the food industry rather than the consumer. Following successful implementation of sodium reformulation policies in other countries such as the United Kingdom and Turkey,^3^ in 2016 the US Food and Drug Administration (FDA) proposed voluntary reformulation targets to reduce sodium content in processed foods and overall population sodium intake by 40% over 10 years. Sodium reduction has been touted as a “best buy” by the World Health Organization (WHO), and our previous investigation estimated it would be very cost‐effective for the United States as a whole.^4^

Food producers have countered that reformulation is a technically difficult and expensive process.^5^ For example, our group recently estimated that following the FDA's proposed voluntary reformulation could cost the industry about $16 billion over 10 years,^4^ or about three‐quarters of a percent of the total revenue of the processed food industry of around $211 billion per year. However, the United Kingdom voluntary initiative resulted in 7% sodium reduction in processed foods,^6^ while the industry continued to grow, so reformulation may be feasible when national policy creates a level playing field for all business. The European Salux project gave examples of how sodium could be substituted in many foods.^6^ Companies like Nestlé, Mars, and General Mills have already responded to the originally proposed FDA voluntary targets by significantly reducing sodium in foods.^4^

An important unanswered question is, what are the costs and benefits of such reformulation to the food industry itself? Millions of Americans work in the US food system, where the private sector subsidizes most health care for employees and their families. While the food industry bears the cost of reformulation, reduced sodium intake among its workers may benefit the corporations, their workers, and their families in the form of reduced health care costs and lower rates of chronic disease, as well as reduced absenteeism and increased productivity.

Given this complex industry dynamic, the overall net impact of the FDA‐proposed policy to the food industry itself is unclear. The present paper is the first to tackle the understudied issue of internal health‐related costs of food policy change for companies and people working in the food system. We conducted a cost‐effectiveness analysis to quantify this impact, accounting for reformulation costs to the food industry and the health benefits to its employees. In this study, we included total national implementation costs, including governmental costs, but included only benefits that fell to individuals who worked in the food system. This investigation was performed as part of the Food‐PRICE (Policy Review and Intervention Cost‐Effectiveness) Project (http://www.food-price.org).

## Methods

### Study Overview

We used and extended our previously validated US IMPACT Food Policy model^7^ to assess the potential health and economic effects of the proposed FDA sodium voluntary reformulation policy on companies and their employees working in the food system, including the overall food system and the subset of the processed food industry, over a 20‐year period (2017‐2036). Please see the Online Appendix, which describe the modeling approach in more detail.

### Microsimulation Model Structure

Ours is a stochastic dynamic microsimulation model that simulates the life course of “close‐to‐reality” synthetic individuals under different policy scenarios. It tracks individual‐level sodium consumption, its impact on systolic blood pressure (SBP), and the subsequent risk of developing CHD, stroke, and death from these or any other cause. The US Sodium Policy model is calibrated to forecasts of CHD, stroke, and any‐other‐cause mortality for the whole US population from 2017 to 2036 (see Online Appendix). Estimates for the ideal minimum level of sodium consumption,^8^ ideal SBP,^9^ the relationship between sodium consumption and SBP, and the relationship between SBP and CHD/stroke mortality,^1^ and between SBP and other mortality,^10^ were derived from meta‐analyses and meta‐regressions. For this study, we assumed that sodium consumption estimated from the National Health and Nutrition Examination Survey (NHANES) using 24‐hour recalls is representative of the US population. An independent validation study with 24‐hour urine collections supports this assumption.^11^ For CHD incidence, we used CHD mortality (ICD‐10 I20–I25) for the United States in 2014,^12^ self‐reported prevalence of CHD from NHANES 2013‐2014,^13^ and the one‐year risk (calculated from 10‐year risk) of CHD for the NHANES 2013‐2014 participants using the Framingham equation^14^ to inform the WHO DisMod II model.^15^ A similar process was used for stroke incidence.^16^, ^17^ DisMod II is a multistate life table model that can estimate the incidence, prevalence, mortality, case fatality, and remission of a disease when information about at least three of these variables is available.

### FDA Sodium Reformulation Scenarios

The FDA's proposed sodium reformulation policy included specific main and upper‐bound sodium concentration targets at two and ten years for 155 food categories.^18^ We considered two policy scenarios:

1. A long‐term compliance scenario, assuming all processed foods will be reformulated to the FDA proposed two‐ and ten‐year sodium targets in time.
2. A short‐term compliance scenario, assuming all processed foods will be reformulated to the two‐year target but with no further reformulation.

We assumed a gradual continuous reformulation to targets and that sodium reduction would be maintained after the end of the policy, for both scenarios.

### Simulated Workforce Population

We used the American Community Survey 2010‐2014 in the IPUMS‐USA database to inform the model with the sociodemographic information (age, sex, race/ethnicity, education, and income) of the food system workforce.^19^ We then used NHANES 2011‐2014 to inform the model with exposures of the US population to sodium and SBP.^20^ Assuming that the food system workforce shares similar exposure patterns with the general US population when adjusted for age, sex, race/ethnicity, education, and income, we produced a close‐to‐reality synthetic population from the synthesis of the two surveys. The statistical framework of this method and its extension to modeling has been described previously.^21^

We used two approaches to define the workforce. One considered the whole food system (around 7.3 million people), which includes people working in food and drink establishments, and a subset of individuals working specifically in the processed food industry (around 1.1 million people) (see NAICS codes in Table 1). We could not find specific information regarding workforce retention in the food industry; therefore, we ran the simulation twice. The first time we simulated a closed cohort of “current” workers at the start of the model. The second time, we simulated an open cohort in which we allowed workers to leave or join the industry as long as the joint distribution of age, sex, race/ethnicity, education, and income remained stable. We report the results of this open cohort as “ever” workers—those who ever worked in the system across the 20 years, including current and former workers.

**Table 1:** Cost‐Effectiveness Model Results for 20 Years, From 2017 to 2036, Long‐Term Compliance Scenario^a^

Model Outputs	Food System Ever Workers	Food System Current Workers	Processed Food Industry Ever Workers	Processed Food Industry Current Workers
NAICS codes, industries modeled	722,000, Food Services and Drinking Places; 445,000, Food and Beverage Stores; 311,000, Food Manufacturing		311,000, Food Manufacturing	
Population modeled (millions)	19.0 (18.7 to 19.2)	7.3 (6.7 to 8.0)	3.1 (3.0 to 3.1)	1.1 (1.0 to 1.2)
Median sodium consumption in 2036 (mg/day)	2,213 (2,204 to 2,221)	2,327 (2,311 to 2,341)	2,243 (2,234 to 2,251)	2,359 (2,343 to 2,375)
Median SBP in 2036 (mmHg)	114 (113.9 to 114.2)	113.2 (112.9 to 113.4)	114.8 (114.6 to 115)	114.4 (114.2 to 114.7)
CVD cases prevented or postponed (undiscounted)	38,700 (21,795 to 65,105)	10,100 (4,700 to 19,400)	7,140 (3,899 to 12,062)	2,020 (960 to 3,742)
CVD deaths prevented or postponed (undiscounted)	3,000 (1,300 to 5,800)	1,200 (300 to 2,500)	600 (260 to 1,101)	200 (60 to 421)
Non‐CVD deaths prevented or postponed (undiscounted)	3,500 (1,900 to 5,700)	1,400 (600 to 2,500)	660 (300 to 1,041)	220 (60 to 400)
QALYs gained (discounted)	180,535 (150,159 to 209,477)	67,411 (54,923 to 80,639)	32,364 (27,114 to 37,415)	11,581 (9,674 to 13,981)
Change in health‐related costs (discounted; billions of dollars)	−5.2 (−8.3 to −3.5)	−1.4 (−2.4 to −0.971)	−0.989 (−1.6 to −0.658)	−0.277 (−0.448 to −0.189)
Change in policy and industry costs (discounted; billions of dollars)	16.6 (6.1 to 35.3)	16.6 (6.1 to 35.3)	16.6 (6.1 to 35.3)	16.6 (6.1 to 35.3)
Total net cost (societal perspective) (discounted; billions of dollars)	11.2 (0.261 to 30.0)	15.1 (4.4 to 33.4)	15.6 (5.0 to 34.2)	16.4 (5.8 to 35.0)
Net monetary benefit (valuing QALYs at $100,000) (discounted; billions of dollars)	6.8 (−12.0 to 18.4)	−8.3 (−27.5 to 2.2)	−12.4 (−31.4 to −1.7)	−15.1 (−34.0 to −4.6)
Incremental cost‐effectiveness ratio—all persons (discounted; 2017 US dollars per QALY)	62,000 (1,000 to 171,000)	224,000 (66,000 to 508,000)	486,000 (148,000 to 1,094,000)	1,404,000 (485,000 to 3,120,000)
Incremental cost‐effectiveness ratio—men (discounted; 2017 US dollars per QALY)	42,000 (−10,000 to 131,000)	165,000 (41,000 to 383,000)	399,000 (121,000 to 933,000)	1,145,000 (401,000 to 2,595,000)
Incremental cost‐effectiveness ratio—women (discounted; 2017 US dollars per QALY)	97,000 (18,000 to 256,000)	331,000 (108,000 to 771,000)	664,000 (220,000 to 1,518,000)	2,077,000 (758,000 to 4,733,000)

Abbreviations: CVD, cardiovascular disease; NAICS, North American Industry Classification System; QALY, quality‐adjusted life years.

a Numbers in parentheses indicate 95% uncertainty intervals.

### Model Structure and Outputs

**Figure 1:**
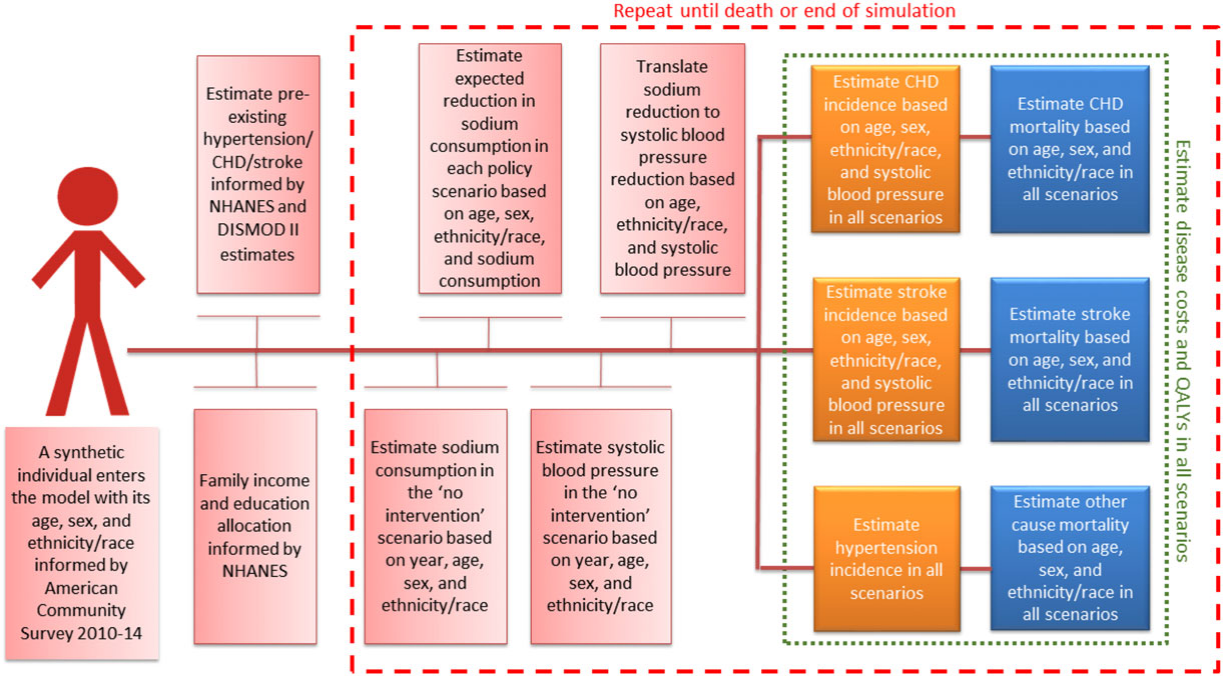
Simplified Model Structure Abbreviations: CHD, coronary heart disease; NHANES, National Health and Nutrition Examination Survey; QALY, quality‐adjusted life year. [Color figure can be viewed at http://wileyonlinelibrary.com]

Figure 1 shows a simplified model structure. The model first simulates the life course of the synthetic individuals under a “business‐as‐usual” scenario. This scenario assumes that the recent trends in sodium exposure, SBP, and CVD mortality will continue in the future. It also assumes that the CVD burden of the simulated workforce is similar to the general US population when adjusted for age, sex, and race/ethnicity. It then simulates the life course of the same individuals under the policy scenarios and compares the outcomes. For each scenario, the model generated the total numbers of relevant events and reports cases and deaths prevented or postponed (CVD or other), QALYs, and disaggregated disease costs. We present the results for adults aged 30 to 84 years from 2017 to 2036 (simulation horizon of 20 years), rounded to the second significant digit.

### Health‐Related Costs and QALYs

The disease medical and productivity costs per person year were derived from the raw data from a report on costs of CVD that was based on the Medical Expenditure Panel Survey (MEPS) 2009‐2013.^22^ Costs include CVD events that are delayed but do not include non‐CVD costs (ie, nonrelevant costs). Productivity costs were split into morbidity (costs from living with disease) and mortality (costs from early death). Productivity costs included workplace, home, and leisure time productivity losses. We estimated informal care costs using published data.^23^, ^24^ Cost inputs were stratified by age and sex and ethnicity/race, except informal care costs.

We calculated QALYs by applying health state utility values (preference weights) to synthetic individuals in the model using published equations that used EQ‐5D‐3L data from MEPS 2000‐2002.^25^ These equations include age, gender, education, income, and comorbidities.

### Sodium Reformulation Industry Costs

Policy costs included total national government outlays to administer and monitor the policy as well as total national industry costs incurred through reformulating products. Government costs of implementation and monitoring were included to account for the full costs of reformulation, even though these costs do not fall to the industry. These costs represented a very small proportion of total costs of reformulation at 1% to 3% depending on the scenario. For industry costs, we used a reformulation cost model developed by Research Triangle Institute (RTI) International under contract with the FDA.^26^ The model accounted for variations in product formula complexity, company size, reformulation type, compliance period, and other factors, which produces a more accurate cost estimate compared to a standard per‐product cost approach. The cost estimates and model equations were developed based on information obtained during two panel meetings conducted by RTI with industry experts. The model uses Universal Product Codes, number of unique product formulas, and unit sales data from 2012. Administrative costs are assumed to occur every year up to year 10, with monitoring and evaluation costs occurring every year after full policy implementation at year three. For the short‐term compliance scenario, we assumed that there would be only one round of reformulation to meet the two‐year targets and that the industry reformulation costs were spread equally from intervention years one to three. For the long‐term compliance scenario, we assumed the industry cost was equal in the two rounds of reformulation (two‐ and ten‐year targets), and divided the costs over the policy implementation years (intervention years one through three for the first round, and intervention years four through ten for the second round). We assumed no policy costs after intervention year 10 but measured health‐related costs over the 20‐year model run.

### Cost‐Effectiveness Analysis

For each scenario and simulated population, the model generated the total numbers of relevant events and reported cases and deaths prevented or postponed (CVD or other), QALYs, and disaggregated disease costs. We present the results for adults aged 30 to 84 in each year from 2017 to 2036 (simulation horizon of 20 years). This age bracket encompasses the window in which hypertension and CVD are most likely to occur and to be preventable through lifestyle change. We evaluated cost‐effectiveness from a societal perspective, including health care costs, as well as productivity, informal care, and industry and governmental costs of reformulation, adhering to the recommendations from the second panel on cost‐effectiveness.^27^ All costs were inflated to 2017 US dollars using the Consumer Price Index, and all costs and QALYS were discounted at a 3% annual rate. Willingness‐to‐pay thresholds were evaluated at $150,000 and $50,000 per QALY, consistent with American College of Cardiology/American Heart Association recommendations.^28^ For net monetary benefit (NMB),^29^ we valued each QALY at $100,000.^30^

### Sensitivity and Uncertainty Analyses

We used probabilistic sensitivity analysis via a second‐order Monte Carlo approach that allowed the estimated uncertainty of different model parameters and population heterogeneity to be propagated to the outputs.^31^ We summarized the output distributions by reporting the medians and 95% uncertainty intervals. Discount rate and willingness‐to‐pay values were included in one‐way sensitivity analysis and were allowed to vary in steps between 0 and 9% and between $50,000 and $150,000, respectively.

## Results

### Sodium Intake

**Figure 2:**
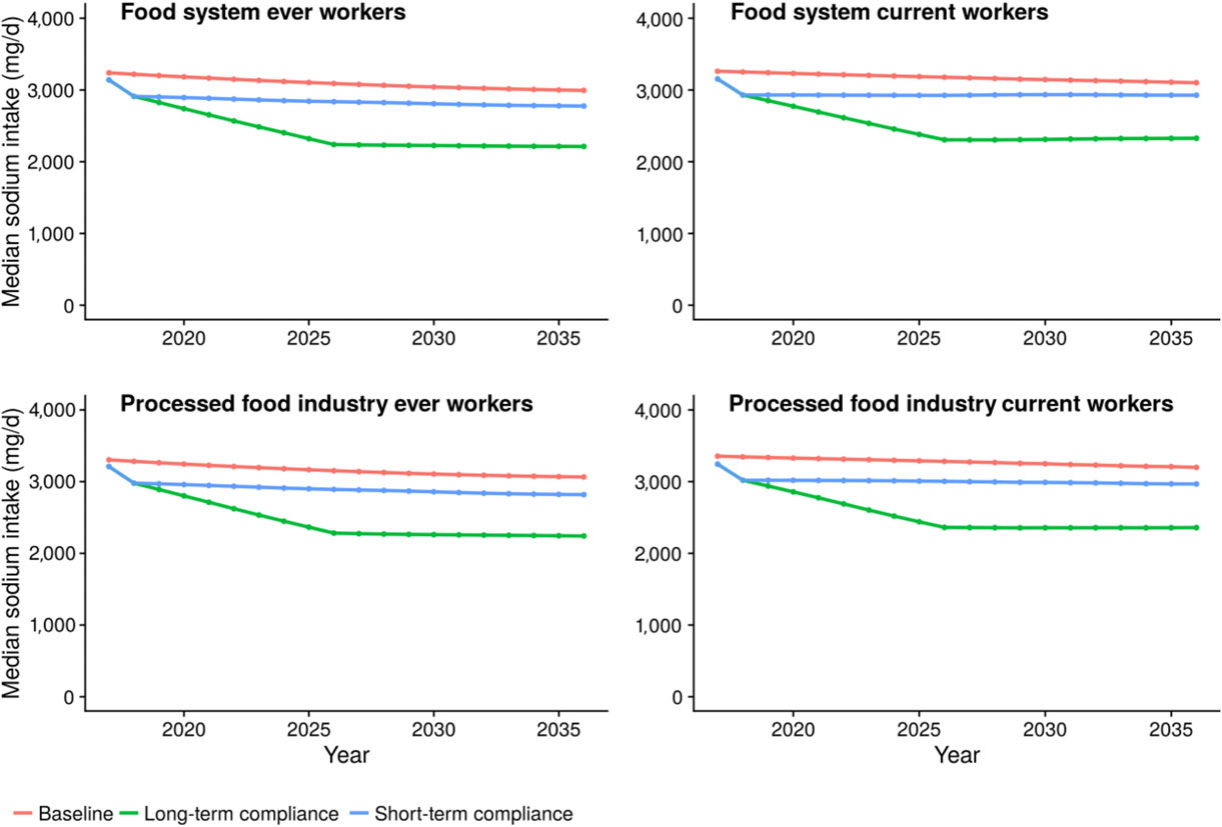
Sodium Consumption (mg/day), by Year, From FDA Sodium Reduction Policy^a^ ^a^Short‐term and long‐term compliance compared with counterfactual baseline of no policy. Cumulative results for 20 years from 2017 to 2036. [Color figure can be viewed at http://wileyonlinelibrary.com]

The number of men and women working in the food system at baseline was similar (9.4 million women, 9.5 million men), but health gains were roughly twice as great in men compared with women (see Online Appendix Table S4). Baseline sodium intake was 3,239 mg/day (95% UI: 3,224‐3,255 mg/day) for food system ever workers in 2017, being marginally higher in processed food ever workers (3,305; 3,288‐3,321). Sodium consumption fell by 32% to 2,213 mg/day (95% UI: 2,204‐2,221 mg/day) for the food system ever workers in the long‐term reformulation scenario, and by 14% to 2,776 mg/day (95% UI: 2,766‐2,786 mg/day) in the short‐term reformulation scenario (Figure 2).

### FDA Sodium Reformulation Industry Costs

Total estimated industry costs of the policy were $7.4 billion (95% UI: $3.3 billion‐$13.6 billion) for the short‐term compliance scenario and $16.6 billion (95% UI: $12.0 billion‐$31.0 billion) for the long‐term compliance scenario. The biggest industry costs were for product reformulation/process modification, project management, and production scale‐up testing. Societal policy costs also included government spending on monitoring and evaluation, which totaled $193 million over the 10 years (see Online Appendix Table S5).

### Health Outcomes and Cost‐Effectiveness for Food System Workers

**Figure 3:**
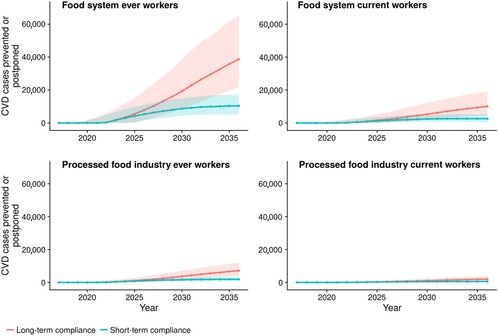
Modeled CVD^a^ Cases Prevented and Postponed, by Year, From FDA Sodium Reduction Policy^b^ ^a^Cardiovascular disease (CVD) includes coronary heart disease and stroke. ^b^ Short‐term and long‐term compliance. Cumulative results for 20 years, from 2017 to 2036. [Color figure can be viewed at http://wileyonlinelibrary.com]

**Figure 4:**
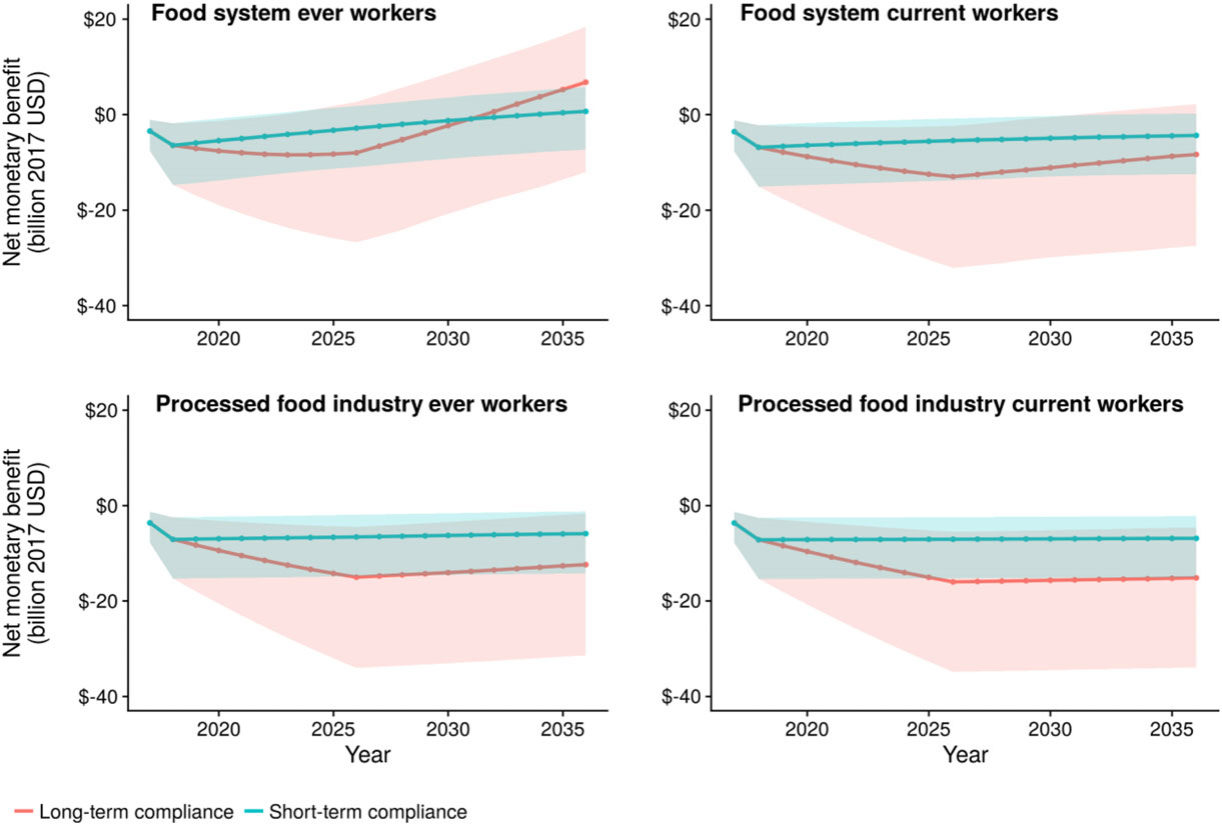
Modeled Net Monetary Benefit,^a^ by Year, From FDA Sodium Reduction Policy^b^ ^a^Net monetary benefit is the total net costs plus health benefits valued at $100,000 per quality‐adjusted life years. ^b^Short‐term and long‐term compliance. Cumulative results for 20 years, from 2017 to 2036. [Color figure can be viewed at http://wileyonlinelibrary.com]

Achieving the sodium reduction targets under the long‐term scenario would prevent or postpone an estimated 38,700 CVD cases among ever food system workers (Figure 3), with health‐related cost savings of approximately $5.2 billion (95% UI: $3.5 billion‐$8.3 billion) and 180,000 (95% UI: 150,000‐209,000) discounted QALYs gained over 20 years (Table 1). The overall ICER, incorporating worker health gains and total health care costs to workers, their employers, and the government, would be around $62,000 per QALY gained (95% UI: $1,500‐$171,000). Overall, with total reformulation costs of $16.4 billion and valuing each QALY at $100,000, this would lead to a net monetary benefit of $6.8 billion (95% UI: −$12 billion to +$18 billion) to the food system, including its workers (Table 1 and Figure 4). When limiting the analysis to a closed cohort of current workers, health‐related cost savings were around $0.47 billion (95% UI: $0.22 billion‐$0.96 billion) with around 67,000 QALYs gained (54,000‐80,000), with an overall ICER of $278,000 and a negative net monetary benefit, that is, a loss of $8.3 billion over 20 years.

For the shorter‐term compliance scenarios of achieving the two‐year reformulation targets only, discounted industry costs were around $7.1 billion and health benefits were roughly a third in magnitude compared to the long‐term scenario (see Online Appendix Table S6). The short‐term compliance scenario was cost‐effective for the ever food system workers with an ICER of $89,000 per QALY (95% UI: $12,000‐$228,000). For current food system workers, the short‐term compliance scenario had a more favorable ICER than the long‐term scenario.

### Health Outcomes and Cost Effectiveness for Processed Food Industry Workers

For ever workers in the processed food industry, an estimated 7,140 CVD cases would be prevented or postponed. Health‐related savings would be approximately $1.0 billion (95% UI: $0.7 billion‐$1.6 billion) with around 32,000 QALYs gained (95% UI: 27,000 to 37,000 QALYs) and an ICER of $486,000 per QALY gained (Table 1). With reformulation costs fixed at $16.4 billion, NMB from the industry perspective would be negative at −$12.4 billion over 20 years. For current workers in the processed food industry only, the QALYs gained and subsequent health‐related cost savings realized would be expected to be approximately one‐quarter (12,000 QALYS, $95 million in health‐related savings). Thirty‐two percent more men than women are ever workers, and 43 percent more men than women are current workers; overall, health gains in men were more than double those for women (see Online Appendix Table S4).

Results for all scenarios by age group (30‐64 and 65‐84) are presented in Online Appendix Table S7.

### Sensitivity Analysis

Input variables were included in probabilistic sensitivity analyses, which had a large effect on the cost‐effectiveness results. For example, among ever workers in the food system, using our long‐term reformulation scenario, NMB from a societal perspective had 95% uncertainty intervals from −$12.0 billion to +$18.4 billion (Table 1, uncertainty intervals in parentheses). The model results are sensitive to the willingness‐to‐pay value of a QALY and to discount rate. For the same population and scenario (ever food system workers, long‐term reformulation), with a high discount rate of 9% and a low QALY valuation of $50,000, the NMB was −$6.4 billion; for a zero discount rate and high QALY valuation of $150,000, the NMB was +$27.5 billion (see Online Appendix Table S8 for results by discount rate and value of a QALY). Many sources of uncertainty are shared between scenarios; therefore, between‐scenario results are not statistically independent and covary to an extent. Therefore, a crossover between UIs for scenarios should not be seen as evidence against statistical significance.

## Discussion

To our knowledge, this is the first study to estimate the potential cost‐effectiveness of the proposed FDA sodium reformulation policy from the perspective of the US food system and its workers. This study suggests that despite the reformulation costs, there may also be several positive financial benefits to the food industry to soften (although not always fully eliminate) the economic cost of pursuing sodium reformulation. When including workers from across the food system, there would be a positive net monetary benefit, due to improved health and reduced health costs. In more conservative scenarios, focusing on workers in the processed food industry alone, the net monetary benefits were negative, but the reformulation costs were still substantially attenuated by the health‐related gains. We included only health gains and cost savings for individuals working in the food system; it is likely that family members who may also benefit may be covered by their employer health insurance, so overall benefits could be greater.

### Implications for Policy

In 2017 Congress temporarily blocked the FDA from implementing voluntary industry targets for sodium reduction. This study provides evidence that considering benefits to the food industry and its workers, this may be a missed opportunity and that such reformulation would yield substantial health gains. If the US government implemented the voluntary targets with encouragement and incentives for the food industry to reformulate, then the benefits of a healthier workforce as outlined here could come to pass. This analysis may help in determining how additional industry benefits such as direct health care savings for their employees and their families could encourage reformulation to improve the health of the whole population more quickly.

Given the direct health harms and economic consequences for health care spending by employers, individuals, and government, excess sodium in the diet may be an example of market failure if food producers are not held to account for the full costs of their products. Health care provision in the United States is at a crossroads, especially given an aging and growing population. All payers of health care—government, employers, and individuals—have great interest, motivation, and aligned incentives to identify upstream cost savings—that is, preventive measures like sodium reduction that will reduce the incidence of health care events rather than reactively treating disease. Rapidly rising health care spending is likely contributing to stagnating wages, reduced GDP growth, and reduced overall employment, as well as reduced government tax returns, while increasing inflation.^32^, ^33^ A program of sodium reduction can be a valuable policy option for helping to counter these trends.

Our analysis considers the overall health gains and costs for the food companies and their employees, as well as associated government health care and implementation costs. For the industry, rising health care premiums mean an increase in part‐time or temporary contracts and reduced wages for employees. CVD‐related morbidity and mortality also negatively affect the industry due to absenteeism, loss of experienced employees, and productivity loss due to workers operating at less than full health—important food industry costs that were not incorporated into our analysis. Much of the CVD associated with sodium may occur after the age of 65, when people are covered by government‐funded Medicare rather than employer health insurance, unless they have retiree coverage offered by some larger companies. Around 20% of people in the food industry do not have health care coverage,^34^ for whom the estimated health care savings will be personal, or governmental if they use Medicaid.

Because consumer taste has been noted as a potential barrier to sodium reduction, gradual national reduction such as under the proposed FDA policy is the optimal approach. Such a gradual reduction across all products in a category will have a negligible effect on preferences (and thus industry sales and profits) as human taste sensitivity adapts within weeks to months to gradually lower sodium levels.^35^ This provides additional justification for a strong national policy, as individual companies may lose market share if they reformulate and others do not.

### Strengths and Limitations

Our study has significant strengths and advancements upon previous work. We utilized a microsimulation model and nationally representative data, increasing confidence in validity and generalizability of our estimates. We model the specific FDA proposal for sodium reformulation, translating changes in sodium content of specific foods to sodium consumption in the US population, rendering our analysis of great relevance and utility to policymakers, industry leaders, and public health advocates. This study is novel in applying a public health economic model with a broad perspective while also taking the perspective of companies and people working in the affected sector of the economy. The model includes both industry and government outlays in terms of policy costs, as well as a range of scenarios and workforce population groups.

Our study has limitations. Specifically, our estimates may be conservative and underestimate the full health and economic benefits of sodium reformulation, for three reasons: First, our baseline scenario assumed that recent observed declines in sodium intake would continue into the future; if recent declines did not continue, the health and economic gains would be larger than those modeled in this analysis. In fact, since we built our model, more recent NHANES data for 2015‐2016 suggests that sodium intake has increased slightly. Second, we evaluated only those diseases mediated through SBP, but decreasing sodium consumption could have beneficial effects upon other health burdens, such as gastric cancer.^36^ And third, we did not include additional benefits achieved with increased potassium intake through substitution of dietary sodium chloride with potassium chloride.^37^ We also do not model how potential differences in health care costs may affect health insurance premiums or how much of these premium costs fall to individuals vs. companies. We have not considered the life cycle of firms in the food industry. The RTI model used to estimate industry reformulation costs may have limitations as well, as estimating true costs to industry can be a challenge.

### Comparison to Other Studies

To our knowledge no similar studies address the impact of sodium reduction on industry; however, parallels may be drawn with the literature around workplace wellness schemes. A meta‐analysis of such schemes found health care costs fall by $3.27 and absenteeism costs fall by $2.73 for every dollar invested in a wellness program.^38^ These benefits mainly accrue directly to individuals but may provide some productivity or health‐related benefits to firms, as well as making companies more attractive to prospective employees. Industry executives may be concerned only with their current employee health costs, but alternatively may think of workers more broadly as being “their people” whose welfare counts highly in their decision making.

## Conclusion

Benefits of implementing the FDA voluntary sodium targets extend to food companies and food system workers, and the value of CVD‐related health gains and cost savings are together greater than the government and industry costs of reformulation. A healthier food system workforce may lead to reduced health insurance premiums and increased employee productivity and retention. Such positive outcomes are of increasing importance and relevance to employers, given that they subsidize the majority of health care in the United States for workers younger than 65 and their families.

## Supporting information

Supplementary Technical and Results Appendix
