# Supplementary material for: FDA Sodium Reduction Targets and the Food Industry: Are There Incentives to Reformulate? Microsimulation Cost‐Effectiveness Analysis
Source: Milbank Q. 2019 Jul 22;97(3):858–80. doi: 10.1111/1468-0009.12402 (PMC6739614; doi:10.1111/1468-0009.12402)
Supplement: Supplementary file 1 — Supplementary Technical and Results Appendix [file MILQ-97-858-s001.pdf]

Supplementary Technical and Results Appendix

This appendix has been provided by the authors to give readers additional information about their work.

Supplement to: FDA sodium reduction targets and the food industry: are there incentives to reformulate?

TABLE OF CONTENTS

Table of Contents 1

Summary of evidence about the risks of excess sodium consumption 3

High-level description of the US SODIUM Policy model 4

Technical information 4

Population module 8

Estimating exposure to risk factors 8

Generating the 'close to reality' synthetic population for the US Sodium Policy model 8

Implementation of individualized risk factor trajectories 9

Demographic and socioeconomic variables 9

Continuous variables 10

Closed and open cohort approaches 12

Disease module 13

Estimating the annual individualized disease risk and incidence 13

Step 1 13

Step 2 13

Step 3 14

Estimating disease incidence at initial simulation year 14

Estimating disease prevalence at initial simulation year 15
Simulating mortality 15
Health economics module 16
Health state utilities 16
Disease costs 16
Policy costs 17
Policy module 19
Modeling the proposed US FDA voluntary sodium reformulation 19
Step 1 19
Step 2 20
Step 3 20
Evidence supporting the choice of baseline scenario 21
Uncertainty and sensitivity analysis 23
Input uncertainty 24
Outputs 25
One-way sensitivity analysis 26
Calibration and Validation 27
INPUTS AND ASSUMPTIONS TABLES 29
ADDITIONAL MODEL RESULTS TABLES 41
References 59

SUMMARY OF EVIDENCE ABOUT THE RISKS OF EXCESS SODIUM CONSUMPTION

Excess dietary sodium consumption has been linked to an increased risk of cardiovascular disease (CVD).[1] For CVD, the excess risk appears to be mainly mediated through the deleterious effect of excess sodium consumption on blood pressure.[2,3] Our methods for evaluating the causality of effects of sodium reduction on BP and BP reduction on CVD have been previously described.[3 Text S1-S3]

The World Health Organization (WHO) and the United States (US) national guidelines recommend a daily sodium intake of less than 2,000 mg/d and 2,300 mg/d, respectively, after assessing the totality of evidence.[4,5] There is some controversy regarding the optimal level of sodium consumption.[6] Some researchers claim that sodium consumption lower than 3,000 mg/d can increase the risk of CVD and overall mortality.[7,8] However, it appears that this argument is based on biased measurement methodology.[9,10] A recent discussion on the subject can be found in Mozaffarian *et al*. who concluded that the optimal level of sodium consumption below which no health gains have been observed is somewhere in the range of 614 mg/d to 2391 mg/d.[3 text S4] In our study we have incorporated the uncertainty around the ideal sodium consumption in our probabilistic sensitivity analysis.

Evidence that directly links sodium risk reversibility to CVD mortality or morbidity outcomes is lacking. A meta-analysis of several randomized control trials that tested low sodium diets was underpowered and therefore inconclusive.[11] In comparison, a plethora of evidence exists supporting the effect of low sodium diet on blood pressure which appears to happen within weeks.[2,3,12] Finally, the cardiovascular risk reversibility of blood pressure has been evident in several randomized control trials and appears to occur within a 5-year period.[13]

HIGH-LEVEL DESCRIPTION OF THE US SODIUM POLICY MODEL

The US Sodium Policy model* is a discrete time dynamic stochastic microsimulation model.[14,15] Within the US Sodium Policy model each unit is a synthetic individual and is represented by a record containing a unique identifier and a set of associated attributes.

For this study, we considered age, sex, race/ethnicity†, education‡, income§, sodium consumption, and systolic blood pressure (SBP). This was matched to the age, sex, race/ethnicity**, education††, income‡‡ distribution of individuals working in the food system from the American Community Survey 2010-2014 in the IPUMS-USE database.[16] A set of stochastic rules is then applied to these individuals, such as the probability of developing coronary heart disease (CHD) or dying, as the simulation advances in discrete annual steps. The output is an estimate of the burden of CHD and stroke, in the synthetic population including both total aggregate change and, more importantly, the distributional nature of the change.

The US Sodium Policy model is a complex model that simulates the life course of synthetic individuals and consists of four modules: The 'population' module, the 'disease' module, the 'health economics' module, and the 'policy' module. We will fully describe the US Sodium Policy model by describing the processes in each of the modules in the following chapters. The description is from an epidemiological rather than technical perspective. Figure S 1 and Figure S 2 depict the logic and structure of the model, respectively. Table S 1 and Table S 2 summarize the sources of the input parameters and the main assumptions and limitations, respectively.

Technical information

The US Sodium Policy model is being developed in R v3.4.0[17] and is currently deployed in a 40-core workstation with 192Gb of RAM running Ubuntu v16.4 server edition. The US Sodium Policy model is built around the R package 'data.table'[18], which imports a new heavily optimized data structure in R. Most functions that operate on a data table have been coded in C to improve performance. Each

---

* This is an extension of the US IMPACT Food policy model. To avoid confusion with previous versions of the model, in this document we will refer to it as 'US Sodium Policy' model.

† Hispanics / non-Hispanic whites / non-Hispanic blacks / other

‡ Less than 9th grade / 9-11th grade (Includes 12th grade with no diploma) / high school graduate/GED or equivalent / some college or AA degree / College graduate or above

§ Based on ratio of family income to poverty: <1.25 / 1.25 – 2 / 2 – 4 / 4+

** Hispanics / non-Hispanic whites / non-Hispanic blacks / other

†† Less than 9th grade / 9-11th grade (Includes 12th grade with no diploma) / high school graduate/GED or equivalent / some college or AA degree / College graduate or above

‡‡ Based on ratio of family income to poverty: <1.25 / 1.25 – 2 / 2 – 4 / 4+

iteration for each scenario is running independently in one of the CPU cores, and the R package ‘foreach’[19] is responsible for the distribution of the jobs and collection of the results. To ensure statistical independence of the pseudo-random number generators running in parallel, the R package ‘doRNG’[20] was used to produce independent random streams of numbers, generated by L'Ecuyer's combined multiple-recursive generator.[21]

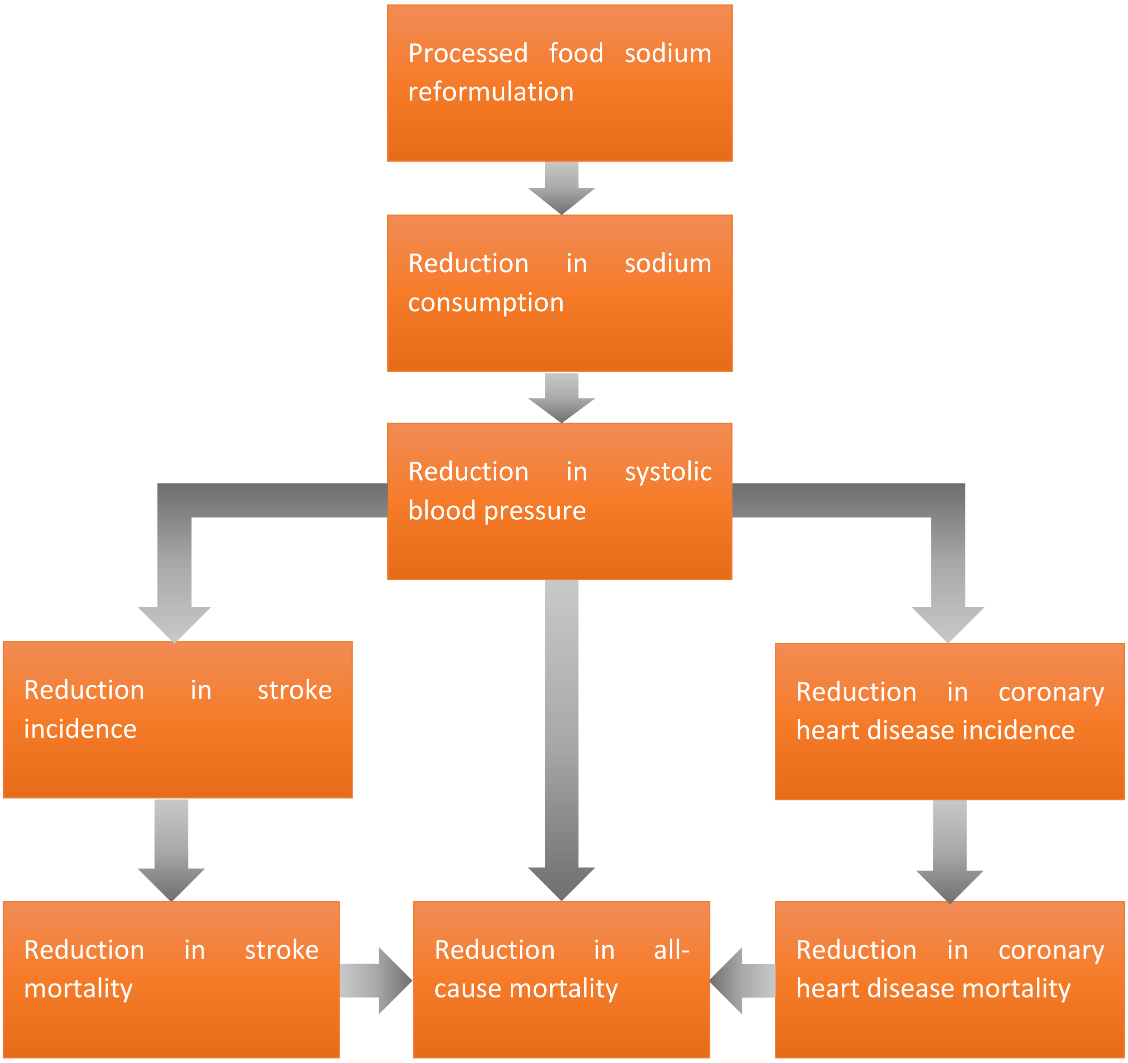

Figure S 1 Model logic. The arrow between reduction in systolic blood pressure and reduction in all-cause mortality does not imply a causal effect of systolic blood pressure on every mortality cause.

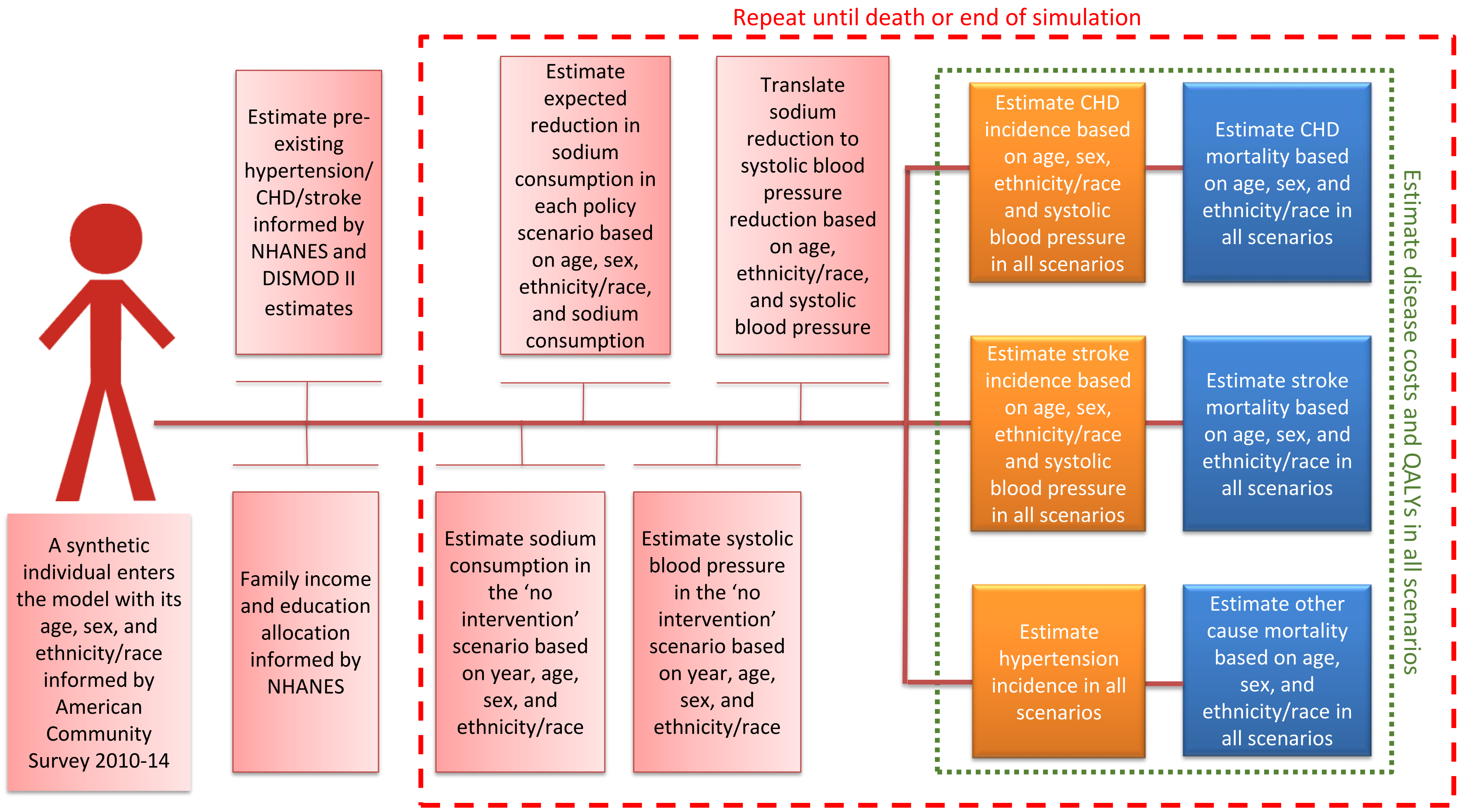

Figure S 2 Simplified model structure. CHD, coronary heart disease; NHANES, National Health and Nutrition Examination Survey; QALY, quality-adjusted life year.

POPULATION MODULE

Synthetic individuals enter the simulation in the initial year (2014 for this study). The number of synthetic individuals that enter the simulation is user-defined and for this study was set to 100,000. The algorithm ensures that the age, sex, race/ethnicity, income, and education distribution of the sample is the same as the worker groups identified in the American Community Survey 2010-2014.[16] The exposures to sodium and SBP are being calculated annually (in simulation time) for each synthetic individual until the simulation horizon is reached, or death occurs.

Estimating exposure to risk factors

The US Sodium Policy model estimates the exposure of the synthetic individual to the modeled risk factors. It is essential the risk profile of each synthetic individual to be similar to the risk profiles that can be observed in the real US population. For this, we first built a 'close to reality' synthetic population of US from which we sampled the synthetic individuals. Then, we used generalized linear models (GLM) for sodium consumption and SBP, to simulate individualized risk factor trajectories for all synthetic individuals.

Generating the 'close to reality' synthetic population for the US Sodium Policy model

The 'close to reality' synthetic population ensures that the sample of synthetic individuals for the simulation is drawn from a synthetic population similar to the real one regarding age, sex, race/ethnicity, and risk factors conditional distributions. In our implementation, we used the same statistical framework originally developed by Alfons *et al.*,[22] and we adapted it to make it compatible with epidemiological principles and frameworks.[23]

In general, this method uses a nationally representative survey of the real population to generate a 'close to reality' synthetic population. Therefore, the method expands the, often small, sample of the survey into a significantly larger synthetic population, while preserving the statistical properties and important correlations of the original survey.

The main advantages over other approaches are: 1) it accounts for the hierarchical structure of the sample design of the original survey, and 2) it can generate trait combinations which were not present in the original survey but are likely to exist in the real population. The second is particularly important because it avoids bias from the excessive repetition of combinations of traits present in the original survey that results from multilevel stratification of a relatively small sample. For example, the original survey may have two 35-year-old male participants, one with SBP of 135 mmHg and the other with an SBP of 140 mmHg and no other 35-year-old male participants with SBP between 135 mmHg and 140 mmHg. Unlike other methodologies, the approach proposed by Alfons *et al.* can produce 35-year-old

male synthetic individuals with an SBP between 135 mmHg and 140 mmHg. This is possible because the synthetic population is produced by drawing from conditional distributions that were estimated from multinomial models fitted in the original survey data. The detailed statistical framework and justification can be found elsewhere.[22]

All the variables of the synthetic population for this study were informed by the National Health and Nutrition Examination Survey 2011–2014 (NHANES1114).[24] The R language for statistical computing v3.4.0 and the R package 'simPop' v0.6.0 were used to implement the method.[25,26] For this study we first generated the demographic variables of the synthetic individuals (age, sex, race/ethnicity). Then, conditional on the demographic variables we generated the educational level variable. We generated income level conditional on the demographic and educational level variables. Finally, we generated sodium[*] consumption on all previously generated variables and SBP conditional on sodium consumption and the demographic variables.

The outcome of the method was to create a synthetic population of 50 million with similar characteristics to the non-institutionalised US population in 2011–2014. Then, for each simulation the model samples 100,000 synthetic individuals from the 50 million population and ensures that their joint age, sex, race/ethnicity, income, and education distribution is proportional to that of the food system (or processed food industry) workforce in the American Community Survey 2010-2014.

Implementation of individualized risk factor trajectories

The US Sodium Policy model only applies the previous process for the initial year of the simulation (2014 for this study). As the simulation evolves, sodium consumption and SBP are recalculated to take into account age and period effects. This feature justifies the classification of the US Sodium Policy model as a dynamic microsimulation. It uses the continuous NHANES series to capture the time trends by age, sex, and race/ethnicity and project them into the future.

Demographic and socioeconomic variables

As the simulation progress in annual circles, the age of the synthetic individuals in the model increase by one year in each loop. Their sex and socioeconomic variables remain stable. Therefore, social mobility is not simulated in the current version of the US Sodium Policy model.

[*] For this study, we assumed that the 24h recall food questionnaire of NHANES is representative of sodium consumption in the US population. An independent validation study with 24h urine collections supports this assumption.[27]

Continuous variables

In the US Sodium Policy model, the value of each continuous risk factor (sodium, SBP) is calculated in a two-step process for each synthetic individual and each projected year. The first step simulates aging effects, while the second step simulates period effects. We follow this approach mainly for two reasons. Firstly, to simulate physiological mechanisms of aging. For example, the increase of SBP due to age-related stiffening of the arteries. Secondly, because the variance of the risk factor distributions increases with age, and we wanted to model this. Below we describe the steps:

Step 1: Instead of tracking the actual continuous risk factor values for the synthetic individuals, we track the percentile ranks[*] of the values by age, sex and race/ethnicity. These percentile ranks remain fixed for each synthetic individual throughout the simulation. In each simulated year, the percentile ranks are converted back to actual risk factor values, by matching the percentile ranks of a sample of the initial synthetic population of same age group, sex, and race/ethnicity.

For example, in 2014 a 30-year-old non-Hispanic black male synthetic individual with SBP of 120 mmHg has an SBP percentile rank of 0.52. Thirty years later, the same synthetic individual has retained his percentile score for SBP. However, his SBP is now calculated to 137.6 mmHg to match the SBP of a 60-year old non-Hispanic black man in 2014 with the same percentile rank of 0.52. Figure S 3 illustrates the previous example. Although individuals retain their percentile for the respective risk factor throughout the simulation (vertical position in Figure S 3), this step remains stochastic because each time this step is implemented a different sample from the synthetic population is drawn. Finally, the distance from the mean for each risk factor is calculated stratified by 5-year age group, sex, and race/ethnicity. For instance, if a synthetic individual has SBP of 140 mmHg and the mean SBP in the respective group of same age group, sex and race/ethnicity is 130 mmHg, the distance from the mean is 140 – 130 = 10 mmHg.

Step 2: We fitted regression models to the continuous NHANES data. For sodium, we used NHANES0914[†], and we fitted a GLM with sodium as the dependent variable and year, age, sex, and race/ethnicity as independent variables (including significant quadratic effects and first order interactions based on Akaike's information criterion (AIC)). For SBP we followed a similar approach,

---

[*] For the percentile rank the formula $R_{percentile} = (R-1)/(n-1)$ is used, where $R_{percentile}$ is the percentile rank and $R = (R_1, \ldots, R_n)$ is the rank vector constructed from a random observation vector $(X_1, \ldots, X_n)$. In this model specifically, vector X is constructed from the subset of the respective continuous risk factor values, by 5-year age group, sex and race/ethnicity, for each year of the simulation.

[†] We used only the recent years of NHANES for two reasons: 1) there was a change in the estimation of sodium intake in NHANES since 2009 which renders older NHANES sodium estimates not immediately compatible with most recent ones; 2) Most importantly, while mean sodium intake was almost constant between years 1999 and 2008 a slow declining trend was obvious in more recent years.

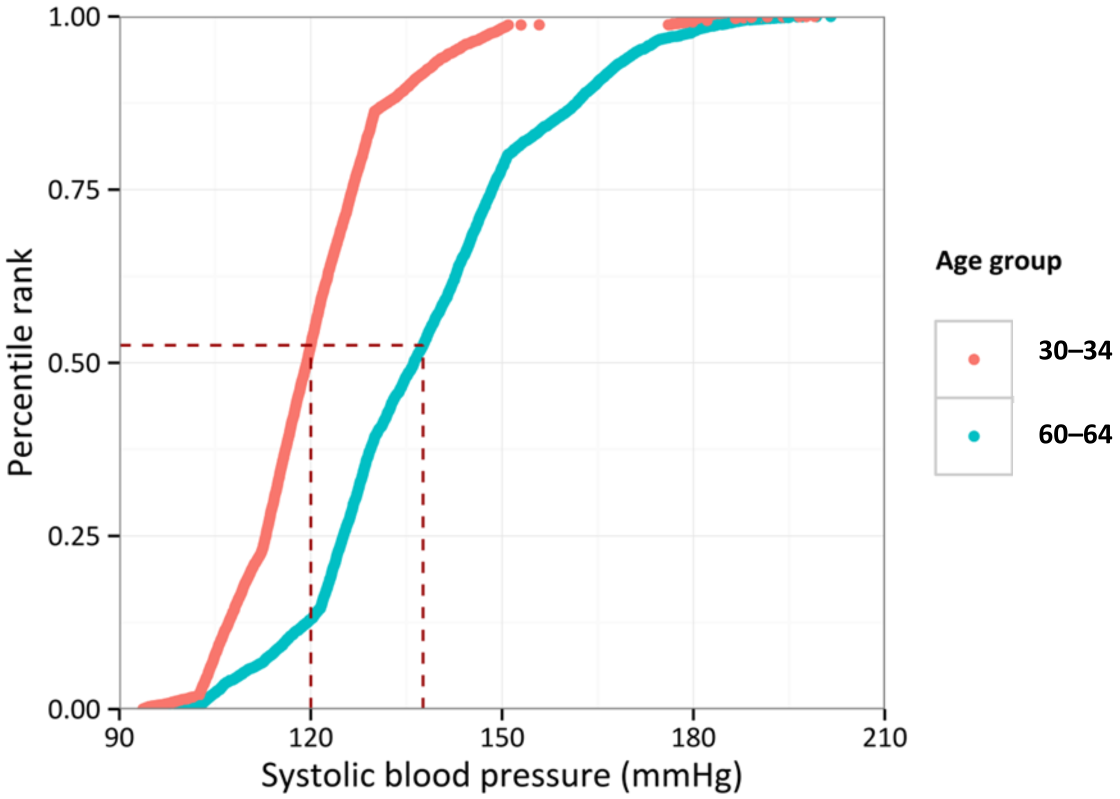

Figure S 3 Plot of the percentile rank against the systolic blood pressure of non-Hispanic black male synthetic individuals for age groups 30–34 and 60–64.

but we used the full range of continuous NHANES9914. For both models, we used a logarithmic link function; therefore, we assumed logarithmic declining time trends for both sodium and SBP. These models are used to predict the mean of the relevant group. These predicted means are added then, to the distances calculated in the previous step. The result is the final value of the relevant risk factor that will be used for risk estimation.

Lag times

All the functions that have been described above for risk factor trajectories include time and age (in years) as one of the independent variables. Therefore, lag times can be potentially calculated on a per risk factor basis. When the 'disease' module of the US Sodium Policy model, uses the exposure to SBP to estimate the risk of a synthetic individual to develop CVD in a specific simulated year, the lag-timed exposure is used. In this study, we assumed that the mean lag time between exposure to high SBP and CVD is five years.[13,28,29] Mean lag times were roughly informed from risk reversibility trials and the median observation times of the cohort studies we used to inform the risk magnitude for SBP. We assumed no lag time between a change in sodium intake and impact on SBP, as this happens within few weeks.[2]

Closed and open cohort approaches

The American Community Survey 2010-2014 provided a snapshot of the joint age, sex, race/ethnicity, income, and education distribution of the food system (and processed food industry) workforce. This distribution remained relatively stable during the 5-year observation period of the survey. In addition, we couldn't find any estimates regarding workforce retention in the sector. Therefore, we did not have much information to simulate individuals leaving and joining the workforce in detail. To overcome this limitation we ran the simulation twice. the first time, simulating a closed cohort of 'current' workers at the start of the model assuming that these workers will remain in the food system (or processed food industry) workforce over the 20-year simulation period.

The second time, simulating an open cohort and easing the assumption that workers will stay in the food system or processed food industry. In this approach, individuals were allowed to leave or join the workforce, under the constraint that the joint age, sex, race/ethnicity, income, and education distribution we observed in the American Community Survey 2010-2014 should remain stable throughout the simulation period. To avoid massive unrealistic movements in and out of the workforce,[*] we added a second constraint to the algorithm to ensure that the minimum necessary number of individuals should move in and out of the workforce.

[*] For example, having all the workforce of a specific age, sex, race/ethnicity, income, and education replaced by new individuals the next simulation year.

DISEASE MODULE

The risk (probability) for each synthetic individual aged 30–84, to develop each of the modeled diseases is estimated conditional on previous exposure to SBP, age, sex, and race/ethnicity. For every simulated year, the model selects synthetic individuals to develop CHD and stroke based on their risk. Finally, the risk of dying from one of the modeled diseases or any other cause is estimated and applied.

Estimating the annual individualized disease risk and incidence

To estimate the individualized annual probability of a synthetic individual to develop a specific disease conditional on his/her relevant risk exposures we follow a 3-step approach. Below we describe the general approach that is used for simulations with more than one risk factors. For this study, only one risk factor was included (high SBP). The implementation of the above method is described in more detail using CHD as an example. The same process is used for both CHD and stroke.

Step 1

The population attributable risk (PAF) is an epidemiological measure that estimates the proportion of the disease attributable to an associated risk factor.[30] It depends on the relative risk associated with the risk factor and the prevalence of the risk factor in the population. In a microsimulation context where exposure to risk factors are known to the individual level and assuming multiplicative risk factors PAF can be calculated with the formula:

$$PAF = 1 - \frac{n}{\sum_{i=1}^{n}(RR_1 * RR_2 * \ldots * RR_k)} ,$$

where n is the number of synthetic individuals in the population, and $RR_{1\ldots k}$ is the relative risks of the risk factors associated with CHD. We calculated PAF based on the formula above stratified by age, sex, and race/ethnicity only in the initial year of the simulation. Consistent with findings from the respective meta-analyses that were used for the US Sodium Policy model (Table S 1), SBP below 110 mmHg, was considered to have a relative risk of 1. All the relative risks were taken from published meta-analyses (Table S 1).

Step 2

The formula below can estimate the incidence of CHD not attributable to the modeled risk factors:

$$I_{Not\ attributable} = I_{Observed} * (1 - PAF)$$

Where $I_{Observed}$ is the CHD incidence and PAF is from Step 1. $I_{Not\ attributable}$ represents CHD incidence if all the modelled risk factors were at optimal levels. The not attributable incidence is calculated by year, age, sex, and race/ethnicity.

To account for future time trend in CHD incidence that is not attributable to the modeled risk factors (in this study SBP), the model updates $I_{Observed}$ every simulated year. For this we assume that half of the forecasted annual change in CHD mortality is attributed to changes in CHD incidence and the other half to changes in CHD case fatality. We based this assumption on observational evidence from England, and modelling studies in the England and the US.[31–34] Furthermore, we included this assumption in our probabilistic sensitivity analysis (page 23).

Step 3

Assuming that $I_{Not\ attributable}$ is the baseline annual probability of a synthetic individual to develop CHD for a given age, sex, and race/ethnicity due to risk factors not included in the model, the individualized annual probability to develop CHD, $\mathbb{P}(\text{CHD} \mid \text{age, sex, race/ethnicity, exposures})$, given his/her risk factors were estimated by the formula:

$$\mathbb{P}(CHD \mid age, sex, \text{race/ethnicity}, exposures) = I_{Not\ attributable} * RR_1 * RR_2 * RR_3 * \ldots * RR_k$$

Where $RR_{1\ \ldots\ k}$ the relative risks that are related to the specific risk exposures of the synthetic individual, same as in step 1.

Estimating disease incidence at initial simulation year

It is evident that for the method above, disease incidence ($I_{Observed}$) in the population, need to be known, at least for the initial year of the simulation. However, the true incidence of CHD (and stroke) in the US, is largely unknown. Several estimates exist nonetheless all have limitations, and the same applies to incidence trends.[35,36] Therefore, for the estimation of CHD and stroke incidence by age, sex, and race/ethnicity we opted for a modelling solution to synthesize all the available nationally representative sources of information and to minimize bias. Specifically, we used CHD mortality (ICD10 I20–I25) for US in 2014,[37] self-reported prevalence of CHD from NHANES1314,[24] and the 1-year risk (calculated from 10 year risk) of CHD for the NHANES1314 participants using the Framingham equation[38] to inform the WHO DisMod II model.[39] DisMod II is a multi-state life table model that can estimate the incidence, prevalence, mortality, case fatality and remission of a disease when information about at least three of these variables is available. A similar approach has been followed by the Global Burden of Disease team and others.[40,41] We considered CHD an incurable chronic disease (i.e. remission rate was set to 0); therefore, the derived DisMod II incidence refers to the first ever episode of CHD excluding any recurrent episodes. For the DisMod II calculations, we assumed that incidence and case-fatality rates had been declining by 2% (relative), over the last 20 years. We used the derived CHD incidence rates by age, sex, and race/ethnicity to inform the US Sodium Policy model. We used the same approach for stroke.[42,43]

Estimating disease prevalence at initial simulation year

For the initial year of the simulation, some synthetic individuals need to be allocated as prevalent cases for each of the modeled diseases. We used DisMod II model estimates for prevalence of CHD and stroke by age, sex, and race/ethnicity. At the beginning of each simulation, the estimated number of prevalent cases are sampled independently from the synthetic individuals in the population with weights proportional to their SBP exposures.

Simulating mortality

All synthetic individuals are exposed to the risk of dying from any of their acquired modeled diseases or any other non-modeled cause in a competing risk framework. The US Sodium Policy model is calibrated to observed CHD, stroke, and any-other-cause mortality for years 2014–2015[37] and mortality forecasts for years 2016–2036. For years after 2015, coherent functional demographic models by sex and race/ethnicity were fitted to the reported CHD, stroke, and any-other-cause mortality rates from years 1999 to 2015,[37] and then were projected to the simulation horizon using the R package 'demography'.[44] Functional demographic models are generalizations of the Lee-Carter demographic model, influenced by ideas from functional data analysis and non-parametric smoothing.[45] The coherent approach ensures that subgroup forecasts do not diverge over time.[46] Finally, we used the observed and forecasted mortality rates to create life tables for each simulated year, by age, sex, race/ethnicity, and disease (CHD, stroke, any-other-cause). We applied the any-other-cause life tables to all synthetic individuals, and the CHD and stroke life tables to prevalent cases of CHD and stroke only, respectively. For the synthetic individual that died of more than one causes in a specific year, a cause was randomly selected to minimize bias.

In reality, hypertensive individuals have a higher risk to die not only of CHD and stroke but from a spectrum of other diseases also. Failure to model this would result in biased estimates in the health economics module (described on page 16) because it would inflate the costs and disutility from hypertension, inappropriately. To account for this and minimize bias the US Sodium Policy model inflates the any-other-cause mortality rates for hypertensive synthetic individuals in the model (Figure S 1 link between SBP and all-cause mortality) while it deflates it for non-hypertensives. The algorithm ensures the total number of hypertensive and non-hypertensive synthetic individuals that die every year from any-other-cause is equal to the defined one in the life table. The algorithm is based on PAF approach, and the relative risk was derived from an individual level meta-analysis by Stringhini *et al*.[47] In this meta-analysis the relative risk of all-cause mortality for hypertensives was 1.31 (1.24–1.38), and the relative risk of non-CVD-non-cancer mortality was 1.29 (1.21–1.38). Hence, we used a relative risk of 1.3 in the US Sodium Policy model.

HEALTH ECONOMICS MODULE

In the previous two modules, the US Sodium Policy model creates synthetic individuals with traits similar to those observed in the US population and tracks their future exposures to sodium and SBP, and important events (first manifestation of CHD and stroke, death from CHD, stroke, or any other cause).

Health state utilities

We calculated the health state utility values (preference weights) using published censored least absolute deviations regression equations which used EQ-5D-3L data from the Medical Expenditure Panel Survey (MEPS) 2000-2002 for all major chronic conditions in the USA, including CHD, stroke, and hypertension.[48] The equation uses the main condition, number of coexisting chronic conditions, age, sex, race, ethnicity, income, and education to estimate the health state utilities of the synthetic individuals every simulated year. We found that the most influential parameter in this equation was the number of coexisting chronic conditions, far exceeding its sampling error. Hence, we ignored the sampling error of this equation, and we calibrated the distribution of the number of coexisting chronic conditions in the synthetic population to the distribution reported by Sullivan *et al*.[48] We further modeled the number of coexisting chronic conditions to increase with age.

Disease costs

The US Sodium Policy model applies CHD, stroke, and hypertension costs to cases of these diseases, during the simulation. These costs are mean estimates by age, sex, and race/ethnicity.

Disease costs per person-year were derived from a report of projections of CVD costs, prepared for the American Heart Association (AHA) by Research Triangle Institute (RTI) International which was based on MEPS data.[49] The AHA report assumed that price increases and new technologies would produce a 2.45% increase in medical costs, above the impact of inflation, demographic change, and disease severity. We assumed an equal annual increase in medical costs. Medical costs per person-year for CHD, stroke, and hypertension were calculated by dividing total medical costs by the number of people with each condition in 2015 and disaggregated by the ratio of the point of service (physician, hospital, prescription, home health, nursing home, and other). The AHA paper included the ratio of medical costs at the point of service for each disease group; the point of service was grouped into physician, hospital, prescription, home health, nursing home, and other.

Productivity costs of morbidity and mortality for CHD and stroke, and hypertension (including workplace productivity and leisure time) were from the same analysis by RTI International and were converted to costs per person-year. For CHD and stroke, we applied morbidity costs to prevalent cases

of CHD and stroke, respectively; we applied mortality costs only to deaths from CHD and stroke. For hypertension, we used the productivity costs not decomposing them into mortality and morbidity costs, because the US Sodium Policy model does not track deaths attributed to hypertension. We assumed that productivity costs would increase by 1.29%, annually.

Informal care costs for stroke were from a study by Joo *et al.*[50], while informal care costs for CHD were based on the ratio of healthcare to informal care costs in Europe from a study by Leal *et al.*[51] We assumed no informal care costs for hypertension alone as we assumed that most would be mediated through CHD and stroke.

Policy costs

The policy costs included:

Government costs to administer and monitor the policy. For administrative costs, because there has been no previous initiative that is both national in scope and precisely about sodium reduction, we used data from two existing sources. First, we acquired cost data from the National Sodium Reduction Initiative (NSRI), led by New York City's Health Department.[52] [*] Second, we acquired cost data from a different FDA policy, new restaurant menu and vending machine labeling regulation, including the cost of outreach, education, review of regulatory issues, developing training for inspectors, and related functions.[53] We used the second data source in the analysis because it generated more conservative (higher-cost) estimates. Monitoring and evaluation cost was obtained through UK FSA's impact assessment and converted to equivalent US dollars[54]. Administrative costs were assumed to occur every year, and monitoring and evaluation costs were assumed to occur every year after full policy implemented in year 3.

Industry costs to reformulate products. Industry costs were calculated using a reformulation cost model developed by the Research Triangle Institute under contract with the FDA.[55] The model accounted for variations in product formula complexity, company size, reformulation type, compliance period and other factors, which produces a more accurate cost estimate compared to a standard per-product cost approach. We calculated the cost of two rounds of reformulation, which corresponded to the FDA's short-term and long-term sodium reduction goals. We assumed the industry cost was equal in the two rounds of reformulation, and divided the costs over the policy implementation years (intervention years 1–3 for the first round, and intervention years 4–10 for the

[*] Sonia Angell, personal communication, Feb 6, 2017

second round). We assumed no policy costs after intervention year 10. All costs were inflated to 2017 dollars and discounted at a 3% rate.

POLICY MODULE

So far, the description of the US Sodium Policy model was for the baseline scenario. The policy module translates the policy scenarios to be modeled by the US Sodium Policy model. Figure S 1 depicts the logic of the model. Changes in sodium consumption are translated into changes in SBP using the meta-regression equation by Mozaffarian *et al.*[3] by age, race, and hypertensive status.[*] The new SBP is used in the disease module, and updated CHD and stroke risks are calculated for every synthetic individual, with new outcomes. Therefore, new life courses for all synthetic individuals are simulated as a result of the modeled policies. When the simulation ends, the model compares all alternative life courses with the baseline one for each synthetic individual and calculates the outputs (page 25).

Modeling the proposed US FDA voluntary sodium reformulation

The US Food and Drug Administration (FDA) in 2016 proposed short-term (2 years) and long-term (10 years) voluntary, category-specific sodium reformulation targets for commercially processed, packaged, and prepared foods. This proposal was designed to support the 2015–2020 US Dietary Guidelines by encouraging food reformulation and new product development.[56] The proposal includes a detailed table with 155 (excluding baby/toddler foods) food categories of commercially processed, packaged, and prepared foods with the baseline (2010) sales-weighted mean sodium concentration the short and long-term target concentrations, and their upper bounds. For example, the 'Blue/Blue-Veined Cheese (Semi-soft)' food category has a baseline sales-weighted mean sodium concentration of 1240mg/100g; short-term target 1180mg/100g; short-term upper bound target 1430mg/100g; long-term target 1050mg/100g; and long-term upper bound target 1340mg/100g;

Separately, FDA also published instruction to link the food categories in the proposal, with the food codes that were used in the 24h recall questionnaires for NHANES0710.[57(p082516)] Unfortunately the linkage was incompatible with more recent NHANES data that we used to prime the synthetic population. Therefore, to model the effect of the proposed policy to the modeled population we developed the algorithm below:

Step 1

We use NHANES0910, and for every participant, we access the 24h recall food questionnaire. The questionnaire contains the amount, food type, and sodium concentration that the participant recalls

[*] We apply this equation only to synthetic individuals with sodium consumption above the optimal level of sodium consumption. Hence, the sodium consumption projection of the baseline scenario is not directly used during this calculation. Only the change in sodium consumption is important and is translated in SBP and health outcomes change.

having consumed the previous day. The FNDDS v5 database was used for the coding of foods.[58] We link these foods with the food categories from the proposed FDA policy. Therefore, we can select which foods in the questionnaires are eligible for reformulation (i.e., their sodium concentration is higher than the proposed FDA targets).[*]

Step 2

Based on the specific scenario assumptions we randomly select eligible foods to be reformulated from the NHANES food questionnaires. We calculate the expected reduction of sodium intake for each NHANES0910 participant, in absolute and relative terms. For this study, we assumed a gradual linear diffusion of the reformulation effect to the population. Once the maximum policy effect was reached, we assumed it would sustain for the rest of the simulation period.

Step 3

We stochastically match each synthetic individual of the US Sodium Policy model with an NHANES0910 participant based on their age (10-year age group), sex, race/ethnicity, and sodium consumption[†]. Then we use the same method described on page 10 to back-project sodium consumption to the year 2010 for all synthetic individuals. Finally, we apply the expected relative sodium reduction of the matched NHANES participant, to the 2010 sodium consumption of the synthetic individuals and we subtract it from the baseline sodium consumption to estimate the net effect of the policy. This net policy effect on every synthetic individual, expressed in a change in sodium consumption every year, is transformed to SBP changes as it was described above. The underlying assumption in this step is that the food composition of US diet has been and will be similar to the one in 2010.

This approach bypasses the incompatibility of the linkage between the FDA proposed policy and more recent NHANES data. It also allows the incorporation of sodium consumption time trends in the calculations and provides enough granularity of the policy effect (by age, sex, race/ethnicity, and sodium consumption) without being too computationally intensive. However, it does not address potential behavioral changes of the population as a result of the reformulation and ignores foods prepared in food outlets and restaurant.

Finally, the linkage between the FDA proposed policy and the FNDDS v5 database was not perfect. Of the 4998 different foods that were linked to 155 food categories in the proposed FDA policy, 132

[*] We assume that the sales-weights are similar to the consumption-weights.

[†] For NHANES participants we used the observed sodium consumption without the expected effect of reformulation.

(2.6%) were not a perfect match, and we manually assigned them to one of the 155 food categories. Table S 3 presents these foods. With the notable exception of imitation cheese and feta cheese in the first row of the table, all other foods contain substantially less sodium compared to products in their food category and were not eligible for reformulation. Recipe conversions and combination codes in the NHANES data were not included in the linkage.

Evidence supporting the choice of baseline scenario

Sodium consumption in NHANES remained stable between 1999 and 2008. Since 2009, an emerging time trend appeared in the data, and mean sodium consumption adjusted for age, sex, and race/ethnicity appears to decrease by almost 18mg per year. Interestingly, the mean sodium concentration of all the foods that were recorded in the FNDDS database, which gets updated every two years, also appears to decline since 2009. Finally, a longitudinal study between 2000 and 2014 using the Nielsen Homescan Consumer Panel data suggests that sodium per capita and food sodium content has been reduced over the period study.[59]

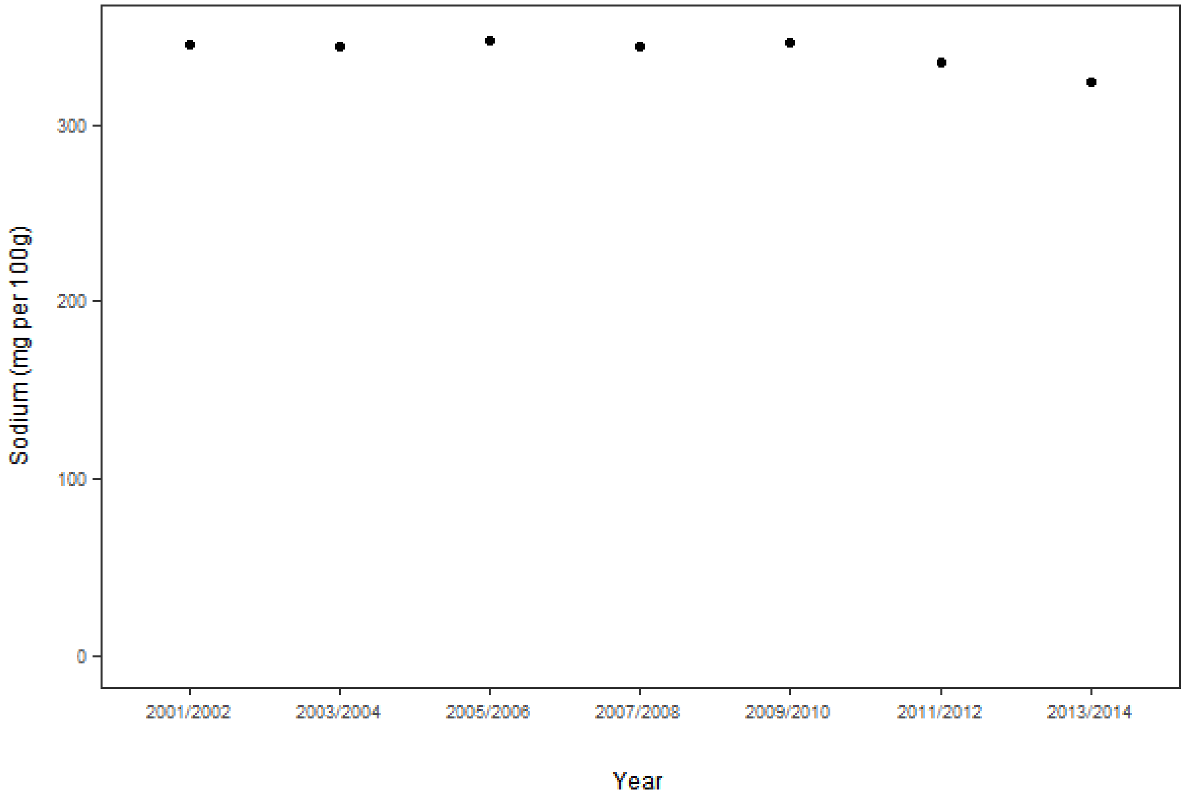

Figure S 4 Mean sodium concentration across all foods recorded in the FNDDS database over time.

Based on the evidence presented above we decided to allow a slowly declining trend in future sodium consumption projections. If the emerging sodium time trend does not continue in the future, will render our results conservative.

UNCERTAINTY AND SENSITIVITY ANALYSIS

The US Sodium Policy model implements a 2nd order Monte Carlo approach to estimate uncertainty intervals (UI) for each scenario.[60,61(p6)] Each simulation, which includes all policy scenarios, runs 2000 times. For each iteration, a different set of input parameters is used by sampling from the respective distributions[*] of input parameters (see page 24), and a different sample of 100,000 synthetic individuals is drawn from the synthetic population of 50 million. Then, the life course[†] of every synthetic individual is simulated for the baseline, and all policy scenarios and the outcomes are collected and summarized for the population. For instance, if a synthetic individual developed CHD at the age of 50 in the baseline scenario and the age of 60 in a policy scenario, this is counted as a CHD case postponed, as a result of the policy. Therefore, all model outputs (cases and deaths prevented or postponed, net utility, net costs, etc. described on page 25) are separately estimated for each iteration, and conditional on the set of model inputs.

The framework allows stochastic uncertainty, parameter uncertainty, and individual heterogeneity to be reflected in the reported UI. The following example illustrates the different types of uncertainty that were considered in the US Sodium Policy model. Let us assume that the annual risk of CHD is 5%. If we apply this risk to all individuals and randomly draw from a Bernoulli distribution with p = 5% to select those who will manifest CHD, we only consider stochastic uncertainty. If we allow the annual risk for CHD to be conditional on individual characteristics (i.e. age, sex, exposure to risk factors), then individual heterogeneity is considered. Finally, when the uncertainty of the relative risks due to sampling errors is considered in the estimation of the annual risk for CHD, the parameter uncertainty is considered. From these three types of uncertainty, only the parameter uncertainty can be reduced from better studies in the future.

The structure of the model is grounded on fundamental epidemiological ideas and well-established causal pathways; therefore, we considered this type of uncertainty relatively small and did not study it. However, the discrete-time nature of the model can potentially introduce bias in cases where the synthetic individual dies more than once within a year, and the model cannot identify which event happened first. As we describe on page 15, to minimize this type of bias we randomly select one of

[*] We assumed log-normal distributions for relative risks and hazard ratios, normal distributions for coefficients of regression equations, generalized beta of the second kind for costs, and PERT distributions for other parameters. The cost sources, except industry reformulation costs, did not include any measures of uncertainty like standard error so an estimate of +/-20% was used for uncertainty analyses, fitted to a generalized beta of the second kind distribution, which can account for the skewness of healthcare costs.[62]

[†] For this study life course actually starts at the age of 30, because it is unlikely that CVD cases and deaths in younger ages can be prevented by sodium intake reduction.

the events to be considered as it happened before all others, whenever these cases arise during the simulation.

Input uncertainty

The sources of uncertainty we considered were:

1. *The sampling error of the baseline sodium intake.* When the model calculates individualized sodium consumptions, it takes into account the sampling error of the regression models that were fitted in the NHANES (see page 10).
2. *The sampling error of the baseline SBP*. Same as above.
3. *The sampling error of the relative risks of SBP on CHD, stroke, and any-other-cause mortality*. We used the reported relative risks and their confidence intervals to construct log-normal (uniform for *any-other-cause mortality*) distributions.
4. *The uncertainty around the lowest exposure to sodium below which no risk is observed.* We used evidence in Mozaffarian *et al.*[3] as parameters for a Pert distribution.
5. *The uncertainty around the lowest exposure to SBP below which no risk is observed*. We used evidence in Singh *et al*.[63]
6. *The uncertainty around the effect of sodium on SBP*. We used the meta-regression equation in Mozaffarian *et al*.[3] Each time the model uses the equation a new set of coefficients was sampled from their respective normal distributions.
7. *The uncertainty around the lag time of SBP exposure and disease outcomes.* The distribution 1 + Binomial(9, (5-1)/9) to vary lag time between 1 and 10 years (median 5 years).
8. *The uncertainty around the true incidence and prevalence rates of CHD and stroke*. We described on page 14 how we used DisMod II to estimate the incidence rate of CHD and stroke. We fitted beta distributions by age, sex, and race/ethnicity assuming the 0.025 percentile to be half of the central estimate, the median the central estimate, and the 0.975 percentile double the central estimate.
9. *The uncertainty of mortality forecasts*. We incorporated the predictive uncertainty of the mortality forecasts to the US Sodium Policy model estimates.
10. *The uncertainty around the assumption that half of the forecasted annual change in CHD and stroke mortality is attributed to changes in CHD and stroke incidence, respectively.* We allowed this assumption to vary, independently for each disease, between 0% and 100% following a uniform distribution.

11. *The uncertainty around which foods will be reformulated.* For policy scenarios that assumed reformulation of less than 100% of eligible products the US Sodium Policy model randomly selected eligible products to be reformulated, in each Monte Carlo iteration.
12. *The uncertainty around the quality of life decrements used to calculate QALY*. The most influential parameter of the equation was the number of coexisting chronic conditions (see page 16). Therefore, we allowed synthetic individuals to have a different number of coexisting chronic conditions in each Monte Carlo iteration.
13. *The uncertainty of all the costs.* The RTI model that was used to estimate the reformulation costs to the industry reported CI. We used these to fit generalized beta of the second kind distributions. For all other costs in the model, we fitted generalized beta of the second kind distribution assuming the 0.2 percentile to be 80% of the central estimate, the median the central estimate, and the 0.8 percentile 120% of the central estimate.

Outputs

We summarize the output distributions of the US Sodium Policy by reporting the medians and 95% uncertainty intervals (UI).

Cases (Deaths) prevented or postponed, by comparing the life course of each specific individual in the baseline scenario with its life course in the policy scenario.

Net utility, by summing the Quality Adjusted Life Years (QALY) through the life course of each specific individual in the baseline scenario and comparing it with the sum of QALY in the policy scenario life course.

Net disease costs, by summing the costs through the life course of each specific individual in the baseline scenario and comparing it with the sum of costs in the policy scenario life course.

Policy costs, by summing the administrative costs, monitoring and evaluation costs, and industry reformulation costs.

All outputs can be stratified by year, age, sex, race/ethnicity, and disease. Moreover, outputs are scaled to the US population (from the 100,000 sample of synthetic individuals).

Costs were analyzed regarding incremental cost-effectiveness ratio (the difference in costs divided by the difference in QALY from the baseline scenario) and net monetary benefit (NMB; incremental net costs plus the value of incremental QALY). For NMB, a central value of $100,000 per QALY gained was

used based on Neumann *et al.*[64] which was varied from $50,000 to $150,000 in a sensitivity analysis. Costs were also presented in a disaggregated 'impact inventory' in line with 2nd US panel recommendations.

From our experience in communicating our results to policymakers and researchers, we realized that they tend to misinterpret 95% UIs as 95% confidence intervals (CI) and overlapping UIs as 'evidence against statistical significance.' This does not apply to our model outputs because the scenarios share common model inputs as explained above and should be treated as 'paired' from a statistical perspective.

One-way sensitivity analysis

For our main analysis, we assumed 100,000 USD willingness to pay per QALY and 3% annual discount rate. We varied these assumptions in the one-way sensitivity analysis, and we present the results for incremental cost-effectiveness ratio and NMB in Table S 9.

CALIBRATION AND VALIDATION

The US Sodium Policy model is calibrated to forecasts of CHD, stroke, and any-other-cause mortality for the whole US population (previously described on page 15). Figure S 5, Figure S 6, and Figure S 7 depict the observed and forecasted mortality that was used for the calibration. We included the uncertainty of the forecasts in our probabilistic uncertainty, and it is propagated in our model estimates.

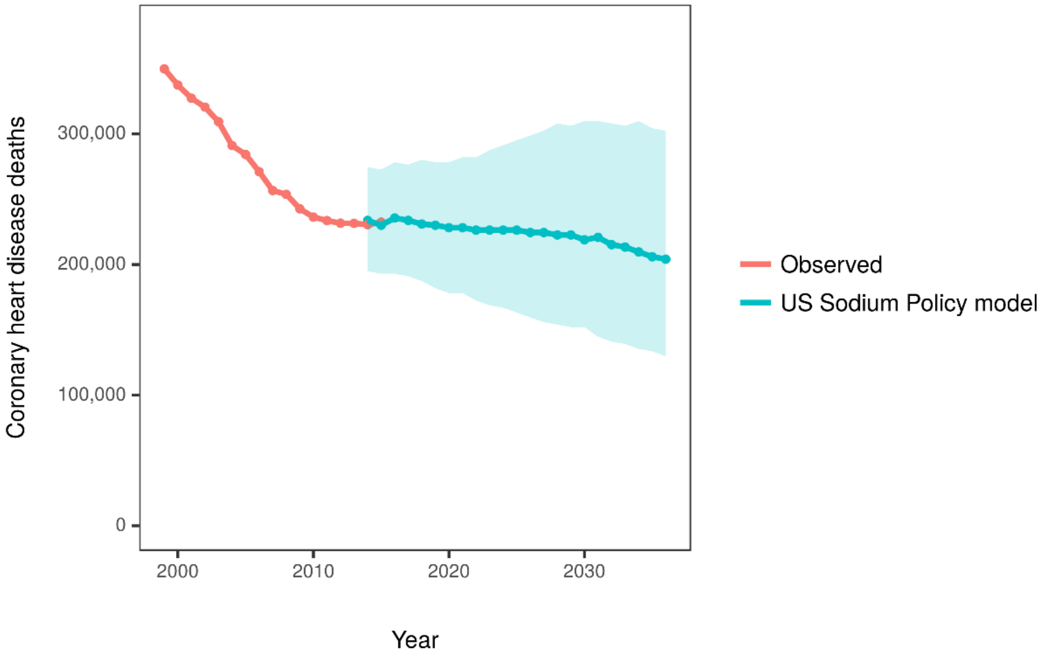

Figure S 5 Observed and forecasted coronary heart disease mortality. US population aged 30 to 84. Shaded areas represent 95% prediction intervals. Source for observed mortality CDC WONDER database.[37]

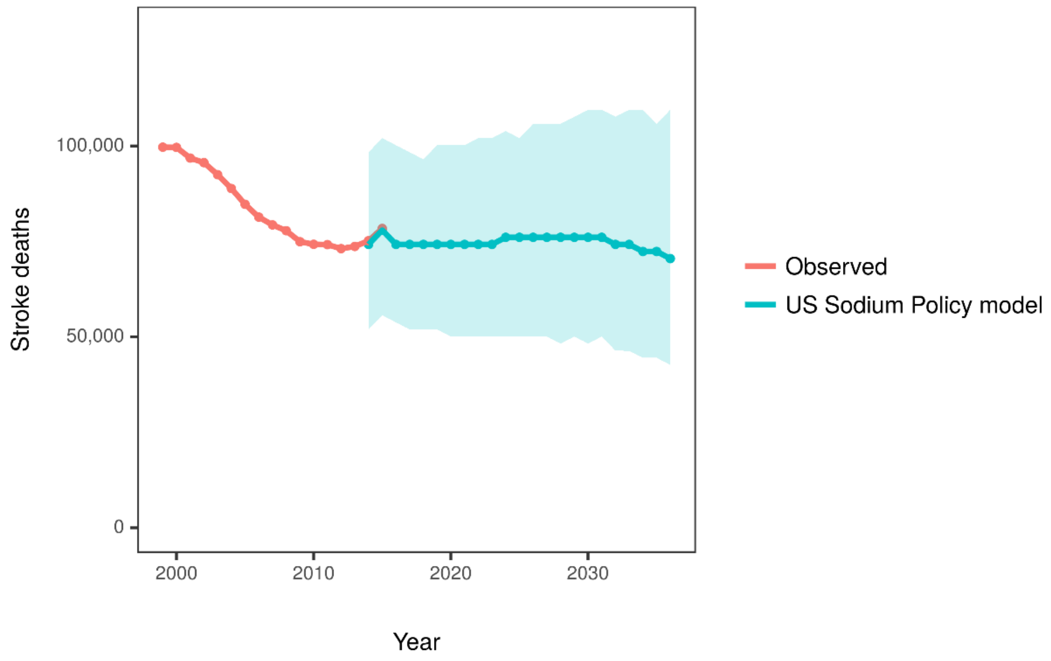

Figure S 6 Observed and forecasted stroke mortality. US population aged 30 to 84. Shaded areas represent 95% prediction intervals. Source for observed mortality CDC WONDER database.[37]

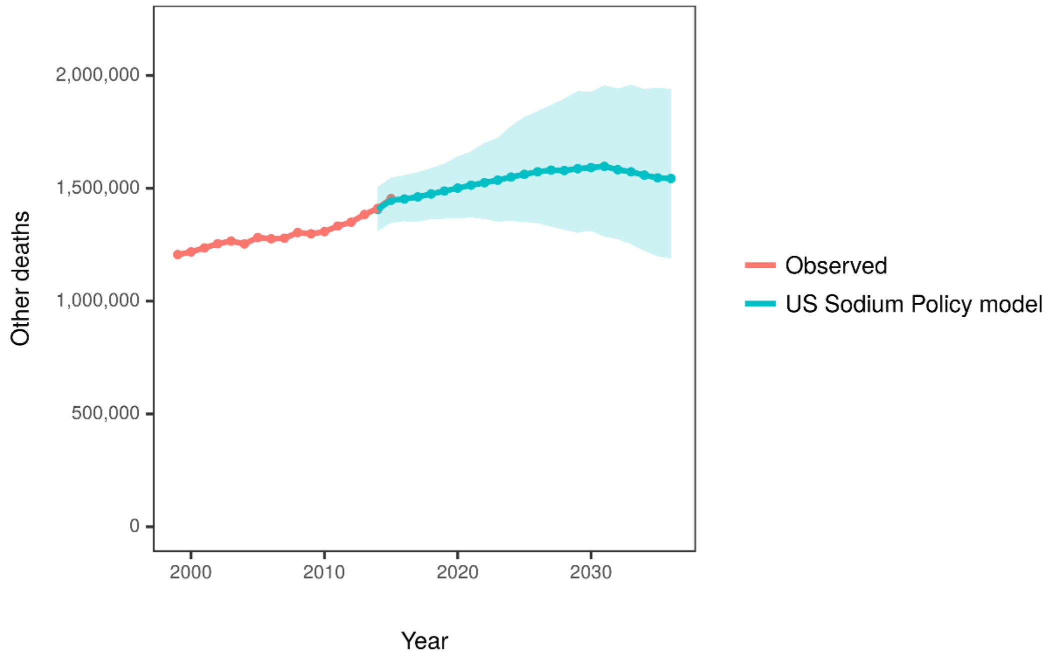

Figure S 7 Observed and forecasted mortality from any-other-cause (excluding coronary heart disease and strokes). US population aged 30 to 84. Shaded areas represent 95% prediction intervals. Source for observed mortality CDC WONDER database.[37]

INPUTS AND ASSUMPTIONS TABLES

Table S 1 The US Sodium Policy model data sources.

Parameter	Outcome	Details	Comments	Source
Population size estimates[16]	Population	Only participants working within the wider food system / processed food industry	Stratified by year, age, sex, bridged-race, and Hispanic origin, income, and education	Ruggles S, Genadek K, Goeken R, Grover J, Sobek M. Integrated Public Use Microdata Series: Version 7.0 [American Community Survey 2010-2014] [Internet]. Minneapolis: University of Minnesota; 2017 [cited 2018 Mar 26]. Available from: https://doi.org/10.18128/D010.V7.0
Mortality[37]	Deaths from CHD, stroke, and any other non-modeled causes	Underlying cause of death 1999–2015	Stratified by year, age, sex, race, ethnicity, and cause of death	United States Department of Health and Human Services (US DHHS), Centers for Disease Control and Prevention (CDC), National Center for Health Statistics (NCHS). Underlying cause of death 1999–2015 on CDC WONDER online database. Data are compiled from data provided by the 57 vital statistics jurisdictions through the Vital Statistics Cooperative Program [Internet]. 2016 [cited 2017 Apr 18];Available from: https://wonder.cdc.gov/ucd-icd10.html
Exposure to sodium[24(pp1999–2014)]	Exposure of individuals	National Health and Nutrition Examination Survey (NHANES)	Anonymized, individual-level data sets. Years 2009–2014.	Centers for Disease Control and Prevention (CDC), National Center for Health Statistics (NCHS). National Health and Nutrition Examination Survey data. Hyattsville, MD: U.S. Department of Health and Human Services, Centers for Disease Control and Prevention, 1999–2014 [Internet]. [cited 2016 Nov 15];Available from: https://wwwn.cdc.gov/nchs/nhanes/ContinuousNhanes/
Exposure to systolic blood pressure[24(pp1999–2014)]	Exposure of individuals	National Health and Nutrition Examination Survey (NHANES)	Anonymized, individual-level data sets. Years 1999–2014.	Centers for Disease Control and Prevention (CDC), National Center for Health Statistics (NCHS). National Health and Nutrition Examination Survey data. Hyattsville, MD: U.S. Department of Health and Human Services, Centers for Disease Control and Prevention, 1999–2014 [Internet]. [cited 2016 Nov 15];Available from: https://wwwn.cdc.gov/nchs/nhanes/ContinuousNhanes/

Parameter	Outcome	Details	Comments	Source
Effect of sodium consumption on systolic blood pressure[3]	Systolic blood pressure change	Meta-analysis/meta-regression of 103 trials	Only trials with duration > 7 days were analyzed.	Mozaffarian D, Fahimi S, Singh GM, *et al*. Global sodium consumption and death from cardiovascular causes. New England Journal of Medicine 2014;371(7):624–34. (Text S1 in the appendix)
Setting reference level of sodium consumption[3]	Ideal sodium consumption below which no risk was considered	Evidence from ecologic studies randomized trials and meta-analyses of prospective cohort studies	Intake levels associated with the lowest risk ranged from 614 to 2391 mg/day. In large, well-controlled, randomized feeding trials, the lowest tested intake for which blood pressure reductions were clearly documented was 1500 mg/day.	Mozaffarian D, Fahimi S, Singh GM, *et al*. Global sodium consumption and death from cardiovascular causes. New England Journal of Medicine 2014;371(7):624–34. (Text S4 in the appendix and Table S3)
Relative risk for systolic blood pressure[47,65]	CHD and stroke (ICD10: I20–I25 and I60–I69)	Pooled analysis of two individual-level meta-analysis	Stratified by age and sex. Adjusted for regression dilution and total blood cholesterol and, where available, lipid fractions (HDL and non-HDL cholesterol), diabetes, weight, alcohol consumption, and smoking at baseline.	Micha R, Peñalvo JL, Cudhea F, Imamura F, Rehm CD, Mozaffarian D. Association between dietary factors and mortality from heart disease, stroke, and type 2 diabetes in the United States. JAMA 2017;317(9):912–24. (eTable5)

Parameter	Outcome	Details	Comments	Source
	Any other mortality (excluding CHD and stroke)	Individual-level meta-analysis of 48 prospective cohort studies	Adjusted for age, sex, race or ethnicity, deprivation, smoking, diabetes, inactivity, alcohol, obesity	Stringhini S, Carmeli C, Jokela M, *et al*. Socioeconomic status and the 25 × 25 risk factors as determinants of premature mortality: a multicohort study and meta-analysis of 1·7 million men and women. The Lancet 2017;389(10075):1229–37. (Figure 4)
Setting reference level of systolic blood pressure[63]	Ideal systolic blood pressure below which no risk was considered	Evidence from evidence from randomized trials of antihypertensive drugs and the Intersalt study	There may be health benefits by lowering systolic blood pressure down to 110mmHg	Singh GM, Danaei G, Farzadfar F, *et al*. The age-specific quantitative effects of metabolic risk factors on cardiovascular diseases and diabetes: a pooled analysis. PLOS ONE 2013;8(7):e65174.
Health state utility values[48]	For CHD, stroke, hypertension, and their combinations	Uses EQ-5D-3L data from the Medical Expenditure Panel Survey (MEPS) 2000-2002	We used the published regression coefficients to estimate utility values by age, sex, race, ethnicity, income, education, and the number of chronic conditions	Sullivan PW, Ghushchyan V. Preference-Based EQ-5D Index Scores for Chronic Conditions in the United States. Medical Decision Making 2006;26(4):410–20. (Tables 2 and 3)
Disease costs[49(pp2015–2035),50,51]	Medical, mortality, and morbidity costs for CHD, stroke, and hypertension	Based on the Medical Expenditure Panel Survey (MEPS)	Stratified by age, sex, and race, adjusted for comorbidities	Khavjou O, Phelps D, Leib A. Projections of cardiovascular disease prevalence and costs: 2015–2035. Technical Report [Internet]. RTI International; 2016 [cited 2017 Jul 10]. Available fromhttps://www.heart.org/idc/groups/heart-public/@wcm/@adv/documents/downloadable/ucm_491513.pdf
	Informal care costs for CHD		Costs were extrapolated for US settings	Leal J, Luengo-Fernández R, Gray A, Petersen S, Rayner M. Economic burden of cardiovascular diseases in the enlarged European Union. Eur Heart J 2006;27(13):1610–9. (Table 5)

Parameter	Outcome	Details	Comments	Source
	Informal care costs for stroke	Difference-in-differences technique to propensity score-matched populations		Joo H, Dunet DO, Fang J, Wang G. Cost of informal caregiving associated with stroke among the elderly in the United States. Neurology 2014;83(20):1831–7. (Table 3)
Government costs to administer the policy[53]		Administration costs for new restaurant menu and vending machine labeling regulation, including cost for outreach, education, review of regulatory issues, developing training for inspectors, and related functions	We assumed sodium reformulation to have same administrative costs	Food and Drug Administration (FDA), Department of Health and Human Services (DHHS). Food and Drug Administration justification of estimates for appropriations committees. Fiscal year 2012 [Internet]. Food and Drug Administration (FDA); 2012 [cited 2017 Jul 10]. Available from: https://www.fda.gov/downloads/AboutFDA/ReportsManualsForms/-Reports/BudgetReports/UCM243370.pdf
Government costs to monitor and evaluate the policy[54]		UK Food Standards Agency impact assessment of UK salt reduction strategy	Costs converted to equivalent US dollars	Collins M, Mason H, O'Flaherty M, Guzman-Castillo M, Critchley J, Capewell S. An economic evaluation of salt reduction policies to reduce coronary heart disease in England: a policy modeling study. Value Health 2014;17(5):517–24.
Industry costs to reformulate products[55(p20)]		Spreadsheet model	The model accounted for variations in product formula complexity, company size, reformulation type, compliance period and other factors	Mary K. Muth, Samantha Bradley, Jenna Brophy, *et al*. Reformulation Cost Model. Contract No. HHSF-223-2011-10005B, Task Order 20. 2015

Table S 2 Key modeling assumptions and limitations. Assumptions about the food system workforce apply for the processed food industry workforce as well

**Population module**
We assumed NHANES to be representative of the US population
We assumed the food system workforce shares similar exposure patterns with general US population when adjusted for age, sex, race/ethnicity, education, and income
We assumed the CVD burden of the food system workforce is similar to the general US population when adjusted for age, sex, and race/ethnicity
**Disease module**
We assumed log-linear exposure-response relationship for SBP with 5-year mean lag time
We only modeled first ever event of CHD and stroke because we focus on primary prevention
For CHD and stroke initial incidence rates (year 2014), we used modeled estimates derived from mortality and NHANES prevalence data
We assumed the non-attributable to SBP incidence rate trends for CHD and stroke, to be 50% of the forecasted mortality rates trends
We assumed that the risk ratios of SBP on CHD and stroke incidence and mortality are equal and SBP is not modifying CHD and stroke survival
We assumed that changes in sodium consumption have an immediate effect on SBP and changes in SBP have a median 5-year time lag to impact the risk of CVD
**Policy module**
We assumed that the recently observed trends in sodium consumption, SBP, and disease-specific mortality would continue in the future (baseline scenario)
We assumed that food system workforce diet has and will have similar food composition since 2010 (the most recent year we can link FDA proposed targets to NHANES)
We did not explicitly model food prepared in restaurants and food outlets
We assumed that individuals would not change sodium consumption behavior because of the policy
We assumed a gradual linear diffusion of the reformulation effect to the population. Once the maximum policy effect was reached, we assumed it would sustain for the rest of the simulation period
**Policy micro-costing**
We estimated the proportion of eligible for reformulation products from the Food and Nutrient Database for Dietary Studies v5
We assumed two rounds of reformulation, one for the 2-year and one for the 10-year target for the long-term compliance. Only the first cycle was considered for the short-term compliance
We assumed that the proportion of sodium reduction during reformulation is not related to the cost of reformulation (echoing the assumption of RTI cost model)

Table S 3 Mismatches between the proposed FDA reformulation policy and the FNDDS v5 database that were handled manually. Except for imitation cheese (1st row), all other foods that were manually assigned to a relevant FDA food category were not eligible for reformulation.

FNDDS food description	FDA food category	Sodium (mg/100g) for the FNDDS food description	Mean sodium in the FDA food category (mg/100g)
Imitation cheese, American or Cheddar type	Feta Cheese (Soft)	1345	1116
Tuna, fresh, raw	Non-Breaded Fish and Other Seafood	45	559
Oysters, raw	Non-Breaded Fish and Other Seafood	85	559
White potato, chips, restructured, reduced fat and reduced sodium	Unflavored Potato and Vegetable Chips	411	550
White potato, chips, unsalted	Unflavored Potato and Vegetable Chips	8	550
White potato, chips, unsalted, reduced fat	Unflavored Potato and Vegetable Chips	8	550
Sweet potato, chips	Unflavored Potato and Vegetable Chips	35	550
Cheese, Cheddar or Colby, low sodium	Cheddar and Colby Cheese (Hard)	21	702
Cheese, Swiss, low sodium	Swiss and Swiss-type Cheese (Hard)	14	263
Butter, stick, unsalted	Butter	11	631
Butter, whipped, tub, unsalted	Butter	11	631
Cucumber salad with creamy dressing	Lettuce/Green Salads: With Additions - Without Dressing	17	339
Artichoke salad in oil	Lettuce/Green Salads: With Additions - Without Dressing	55	339
Cookie, fruit, baby	Toddler Meals and Entrees	9	226
Oatmeal cereal with fruit, baby food, dry, instant, toddler	Toddler Meals and Entrees	0	226
Macaroni, tomatoes, and beef, baby food, junior	Toddler Meals and Entrees	35	226
Macaroni with beef and tomato sauce, baby food, toddler	Toddler Meals and Entrees	35	226
Margarine, stick, unsalted	Margarine and Vegetable Oil Spreads	2	670
Margarine, tub, unsalted	Margarine and Vegetable Oil Spreads	28	670

FNDDS food description	FDA food category	Sodium (mg/100g) for the FNDDS food description	Mean sodium in the FDA food category (mg/100g)
Margarine-like spread, tub, unsalted	Margarine and Vegetable Oil Spreads	2	670
Cookie, fruit, baby food	Baby/Toddler Snacks: Cookies/Biscuits	9	326
Fruit dressing, made with honey, oil, and water	Salad Dressing	2	867
Milk, vinegar, and sugar dressing	Salad Dressing	47	867
Peppers, hot, cooked, from frozen, fat not added in cooking	Frozen Vegetables and Legumes	7	254
Turnip greens, canned, low sodium, cooked, fat not added in cooking	Canned Vegetables	29	267
Carrots, canned, low sodium, fat not added in cooking	Canned Vegetables	34	267
Peas and carrots, canned, low sodium, fat added in cooking	Canned Vegetables	16	267
Peas and carrots, canned, low sodium, fat not added in cooking	Canned Vegetables	4	267
Sweet potato, canned, NS as to syrup	Canned Vegetables	44	267
Tomatoes, canned, low sodium	Canned Vegetables	10	267
Beans, string, green, canned, low sodium, NS as to fat added in cooking	Canned Vegetables	21	267
Beans, string, green, canned, low sodium, fat not added in cooking	Canned Vegetables	2	267
Beans, string, green, canned, low sodium, fat added in cooking	Canned Vegetables	21	267
Beets, canned, low sodium, fat not added in cooking	Canned Vegetables	21	267
Corn, yellow, canned, low sodium, fat not added in cooking	Canned Vegetables	1	267
Corn, yellow, canned, low sodium, fat added in cooking	Canned Vegetables	19	267
Peas, green, canned, low sodium, fat not added in cooking	Canned Vegetables	9	267
Peas, green, canned, low sodium, fat added in cooking	Canned Vegetables	21	267
Peppers, hot, cooked, from canned, fat not added in cooking	Canned Vegetables	7	267
Peppers, hot, cooked, from canned, fat added in cooking	Canned Vegetables	7	267

FNDDS food description	FDA food category	Sodium (mg/100g) for the FNDDS food description	Mean sodium in the FDA food category (mg/100g)
Pimiento	Canned Vegetables	17	267
Mixed vegetables (corn, lima beans, peas, green beans, and carrots),	Canned Vegetables	26	267
Mixed vegetables (corn, lima beans, peas, green beans, and carrots),	Canned Vegetables	42	267
Cucumber pickles, dill, reduced salt	Pickled Vegetables	18	433
Carrot juice	Vegetable Juice	66	235
Tomato juice, low sodium	Vegetable Juice	10	235
Tomato and vegetable juice, mostly tomato, low sodium	Vegetable Juice	70	235
Mixed vegetable juice (vegetables other than tomato)	Vegetable Juice	63	235
Celery juice	Vegetable Juice	91	235
Aloe vera juice	Vegetable Juice	2	235
Vegetable and fruit juice blend, 100% juice, with high vitamin C plus	Vegetable Juice	29	235
White potato, boiled, without peel, canned, low sodium, fat not added in	Potato Side Dishes	5	315
Almond paste (Marzipan paste)	Nut/Seed Butters and Pastes	9	335
Peanut butter, low sodium	Nut/Seed Butters and Pastes	17	335
Peanut butter, reduced sodium, and reduced sugar	Nut/Seed Butters and Pastes	203	335
Almonds, unroasted	Nuts and Seeds	1	366
Almonds, dry roasted, without salt	Nuts and Seeds	3	366
Brazil nuts	Nuts and Seeds	3	366
Cashew nuts, roasted, without salt	Nuts and Seeds	13	366
Cashew nuts, dry roasted, without salt	Nuts and Seeds	16	366
Chestnuts, roasted	Nuts and Seeds	2	366

FNDDS food description	FDA food category	Sodium (mg/100g) for the FNDDS food description	Mean sodium in the FDA food category (mg/100g)
Filberts, hazelnuts	Nuts and Seeds	0	366
Peanuts, roasted, without salt	Nuts and Seeds	6	366
Peanuts, dry roasted, without salt	Nuts and Seeds	6	366
Pecans	Nuts and Seeds	0	366
Pine nuts (Pignolias)	Nuts and Seeds	2	366
Walnuts	Nuts and Seeds	2	366
Walnuts, honey-roasted	Nuts and Seeds	20	366
Nut mixture with dried fruit and seeds	Nuts and Seeds	7	366
Pumpkin and/or squash seeds, hulled, unroasted	Nuts and Seeds	7	366
Pumpkin and/or squash seeds, hulled, roasted, without salt	Nuts and Seeds	18	366
Sunflower seeds, hulled, unroasted	Nuts and Seeds	9	366
Sunflower seeds, hulled, roasted, without salt	Nuts and Seeds	3	366
Sesame seeds	Nuts and Seeds	39	366
Flax seeds	Nuts and Seeds	30	366
Chicken or turkey soup, cream of, canned, reduced sodium, made with	Canned, Condensed Soup	185	358
Chicken rice soup, canned, reduced sodium, prepared with water or	Canned, Condensed Soup	164	358
Tomato soup, canned, reduced sodium, prepared with water	Canned, Condensed Soup	13	358
Tomato soup, canned, reduced sodium, prepared with milk	Canned, Condensed Soup	33	358
Mushroom soup, cream of, canned, reduced sodium, NS as to made with	Canned, Condensed Soup	207	358
Beer soup, made with milk	Canned, Ready-to-Eat Soup	24	311
Beef and mushroom soup, canned, low sodium	Canned, Ready-to-Eat Soup	25	311

FNDDS food description	FDA food category	Sodium (mg/100g) for the FNDDS food description	Mean sodium in the FDA food category (mg/100g)
Clam chowder, New England, canned, reduced sodium, ready-to-serve	Canned, Ready-to-Eat Soup	194	311
Bean and ham soup, canned, reduced sodium, prepared with water or	Canned, Ready-to-Eat Soup	187	311
Chicken noodle soup, canned, low sodium, ready-to-serve	Canned, Ready-to-Eat Soup	173	311
Chicken noodle soup, canned, reduced sodium, ready-to-serve	Canned, Ready-to-Eat Soup	193	311
Soup, mostly noodles, reduced fat, reduced sodium	Canned, Ready-to-Eat Soup	212	311
Tomato soup, canned, low sodium, ready-to-serve	Canned, Ready-to-Eat Soup	33	311
Vegetable soup, canned, low sodium, prepared with water or ready-to-	Canned, Ready-to-Eat Soup	198	311
Minestrone soup, canned, reduced sodium, ready-to-serve	Canned, Ready-to-Eat Soup	215	311
Vegetable noodle soup, canned, reduced sodium, prepared with water or	Canned, Ready-to-Eat Soup	196	311
Vegetable soup, made from dry mix, low sodium	Dry Mix Soup	194	3342
Chicken broth, canned, low sodium	Shelf Stable Liquid Broth and Stock	30	247
Soy sauce, reduced sodium	Soy Sauce	3333	5637
Cheese, processed, American or Cheddar type, low sodium	Processed Cheese/Cheese Food (Semi-soft)	7	1433
Lemon-butter sauce	Asian-style Sauce	3	1814
Fruit sauce	Asian-style Sauce	62	1814
Tomato catsup, low sodium	Condiments	20	842
100 % Natural Cereal, with oats, honey and raisins, Quaker	Ready-to-Eat Cereal, Flakes	54	492
Wheat germ, plain	Ready-to-Eat Cereal, Flakes	4	492
Crispy Brown Rice Cereal	Ready-to-Eat Cereal, Puffed	12	529
Froot Loops Cereal Straws	Ready-to-Eat Cereal, Puffed	49	529

FNDDS food description	FDA food category	Sodium (mg/100g) for the FNDDS food description	Mean sodium in the FDA food category (mg/100g)
Kashi, Puffed	Ready-to-Eat Cereal, Puffed	10	529
Malt-O-Meal Puffed Wheat	Ready-to-Eat Cereal, Puffed	5	529
100% Natural Cereal, plain, Quaker	Ready-to-Eat Cereal, Puffed	50	529
Wheat, puffed, plain	Ready-to-Eat Cereal, Puffed	5	529
Kashi cereal, NS as to ready to eat or cooked	Prepared Cooked Cereal	10	178
Cornstarch with milk, eaten as a cereal (2 tbsp cornstarch in 2-1/2 cups	Dry Mix Instant Cereal	46	221
Nestum cereal	Dry Mix Instant Cereal	35	221
Flour and water patty	White Bread	4	464
Tortilla, corn	Tortillas and Wraps	45	619
Crackers, saltine, low sodium	Crackers	198	757
Crackers, saltine, fat-free, low sodium	Crackers	716	757
Crackers, toast thins (rye, wheat, white flour), low sodium	Crackers	190	757
Cracker, 100% whole wheat, low sodium	Crackers	186	757
Cracker, snack, low sodium	Crackers	216	757
Cracker, cheese, low sodium	Crackers	458	757
Cracker, snack, low fat, low sodium	Crackers	292	757
Crispbread, wheat, low sodium	Crackers	324	757
Cracker, multigrain, salt-free	Crackers	73	757
Crispbread, rye, low sodium	Crackers	453	757
Crackers, matzo	Crackers	0	757
Cake made with glutinous rice	Cake	12	332

FNDDS food description	FDA food category	Sodium (mg/100g) for the FNDDS food description	Mean sodium in the FDA food category (mg/100g)
Pastry, fruit-filled	Pastries, Pie, and Cobbler	8	251
Ham, sliced, low salt, prepackaged or deli, luncheon meat	Deli Meats - Ham	969	1164
Beef, sandwich steak (flaked, formed, thinly sliced)	Deli Meats - Beef	74	1055
Turkey or chicken breast, low salt, prepackaged or deli, luncheon meat	Deli Meats -Turkey/Chicken	772	1030
Ground beef patty, cooked (for fast food sandwiches)	Deli Meats - Loaves/Mixtures	77	1164
Frankfurter or hot dog, low salt	Frankfurters, Hot Dogs, and Bologna	314	1063
Bologna, beef, lower sodium	Frankfurters, Hot Dogs, and Bologna	682	1063
Pork bacon, smoked or cured, lower sodium	Uncooked Bacon	1030	
Cheese, Mozzarella, low sodium	Pasta Filata Cheese (Soft)	16	668

ADDITIONAL MODEL RESULTS TABLES

Tables S4 to S9 show additional model results that are not in the main paper.

Table S 4. Results by Sex

Model outputs	Sex	**Food System - Ever**	**Food System - Ever**	**Food System - Current**	**Food System - Current**	**Processed Food - Ever**	**Processed Food - Ever**	**Processed Food - Current**	**Processed Food - Current**
		Long-term compliance	Short-term compliance	Long-term compliance	Short-term compliance	Long-term compliance	Short-term compliance	Long-term compliance	Short-term compliance
Population	Men	9,423,300 (9,260,090 to 9,555,760)	9,420,200 (9,257,090 to 9,553,720)	3,639,150 (3,285,290 to 3,978,510)	3,637,700 (3,284,000 to 3,977,410)	1,760,060 (1,726,380 to 1,788,870)	1,759,500 (1,725,760 to 1,788,110)	656,300 (596,139 to 714,902)	656,140 (595,959 to 714,643)
	Women	9,529,550 (9,409,290 to 9,638,210)	9,528,800 (9,408,190 to 9,637,220)	3,707,950 (3,374,080 to 4,026,400)	3,707,050 (3,373,580 to 4,025,820)	1,330,820 (1,310,400 to 1,348,280)	1,330,740 (1,310,260 to 1,348,140)	459,600 (420,120 to 499,121)	459,580 (420,098 to 499,100)
Median sodium consumption in 2036 (mg/d)	Men	2,619.93 (2,606.14 to 2,633.46)	3,318.76 (3,300.36 to 3,336.77)	2,758.99 (2,742.78 to 2,774.43)	3,519.85 (3,496.86 to 3,542.49)	2,595.06 (2,581.09 to 2,608.41)	3,285.06 (3,265.28 to 3,302.49)	2,716.39 (2,698.34 to 2,733.58)	3,461.78 (3,435.14 to 3,487.43)
	Women	1,876.99 (1,867.05 to 1,886)	2,379.54 (2,367.53 to 2,390.73)	1,959.89 (1,944.48 to 1,973)	2,497.37 (2,478.13 to 2,516.77)	1,847.25 (1,837.38 to 1,856.62)	2,345.76 (2,333.41 to 2,356.88)	1,904.73 (1,889.84 to 1,917.52)	2,428.63 (2,409.18 to 2,446.26)
Median SBP in 2036 (mmHg)	Men	117.2 (117 to 117.4)	118.4 (118.2 to 118.6)	117.5 (117.2 to 117.7)	118.6 (118.3 to 118.8)	117.3 (117 to 117.5)	118.5 (118.3 to 118.7)	117.5 (117.3 to 117.8)	118.7 (118.4 to 118.9)
	Women	110.1 (109.8 to 110.5)	111 (110.7 to 111.2)	108.1 (107.7 to 108.4)	108.8 (108.5 to 109)	110.5 (110.2 to 110.9)	111.4 (111.1 to 111.6)	108.8 (108.4 to 109.1)	109.5 (109.2 to 109.8)
CVD cases prevented or postponed	Men	25,500 (14,000 to 41,902.5)	4,300 (900 to 7,902.5)	6,600 (2,897.5 to 12,500)	900 (-800 to 2,702.5)	5,160 (2,860 to 8,581)	920 (239 to 1,861)	1,480 (680 to 2,741)	300 (-21 to 680)
	Women	13,200 (7,000 to 24,600)	6,000 (3,097.5 to 10,602.5)	3,500 (1,597.5 to 7,102.5)	1,600 (500 to 3,402.5)	2,020 (1,000 to 3,682)	940 (420 to 1,661)	520 (220 to 1,080)	240 (80 to 560)
CVD deaths prevented or postponed	Men	2,000 (800 to 4,000)	600 (0 to 1,600)	750 (200 to 1,700)	200 (0 to 800)	440 (159 to 841)	140 (0 to 360)	140 (40 to 340)	40 (0 to 160)
	Women	1,000 (300 to 2,100)	500 (0 to 1,200)	400 (0 to 1,100)	200 (0 to 700)	160 (40 to 341)	80 (0 to 200)	60 (0 to 141)	20 (0 to 100)
Non-CVD deaths prevented or postponed	Men	2,200 (1,000 to 3,702.5)	100 (0 to 1,100)	700 (100 to 1,600)	100 (0 to 600)	480 (200 to 781)	40 (0 to 240)	160 (20 to 300)	20 (0 to 120)
	Women	1,300 (500 to 2,300)	500 (0 to 1,100)	600 (197.5 to 1,300)	200 (0 to 600)	180 (40 to 380)	60 (0 to 180)	60 (0 to 180)	20 (0 to 81)

Model outputs	Sex	**Food System - Ever**	**Food System - Ever**	**Food System - Current**	**Food System - Current**	**Processed Food - Ever**	**Processed Food - Ever**	**Processed Food - Current**	**Processed Food - Current**
		Long-term compliance	Short-term compliance	Long-term compliance	Short-term compliance	Long-term compliance	Short-term compliance	Long-term compliance	Short-term compliance
Life years gained	Men	12,100 (5,397.5 to 21,302.5)	6,100 (1,600 to 12,800)	6,000 (2,400 to 11,905)	3,050 (300 to 7,602.5)	1,840 (600 to 3,520)	920 (79 to 2,084)	680 (100 to 1,640)	320 (0 to 1,040)
	Women	29,300 (15,100 to 44,205)	8,050 (0 to 17,702.5)	10,550 (4,100 to 19,200)	2,800 (0 to 8,900)	6,160 (3,459 to 9,506)	1,740 (0 to 4,020)	2,160 (960 to 3,760)	560 (0 to 1,600)
Discounted QALYs gained (million)	Men	0.113206m (0.0941519m to 0.132812m)	0.0321119m (0.0213289m to 0.0438018m)	0.0434167m (0.0339412m to 0.0536998m)	0.0132506m (0.00788525m to 0.0194223m)	0.0221828m (0.0185961m to 0.0261049m)	0.00642946m (0.00431455m to 0.00863614m)	0.00841539m (0.00670177m to 0.0103014m)	0.00266606m (0.00169531m to 0.0037432m)
	Women	0.067779m (0.0491239m to 0.0833718m)	0.0306041m (0.0226726m to 0.0379584m)	0.0239264m (0.0176941m to 0.031203m)	0.0113685m (0.00842396m to 0.0154044m)	0.0102792m (0.00707395m to 0.0125623m)	0.00474859m (0.00344004m to 0.00602007m)	0.00324891m (0.00219714m to 0.00411497m)	0.00156967m (0.00111252m to 0.00210256m)
Change in health-related costs:	Men	-3,509.88m (-5,788.28m to -2,425.95m)	-989.259m (-1,853.39m to -552.709m)	-977.583m (-1,774.8m to -656.846m)	-256.694m (-648.072m to -44.2127m)	-725.515m (-1,225.55m to -492.247m)	-212.711m (-393.81m to -123.371m)	-211.313m (-345.002m to -140.701m)	-63.6484m (-120.348m to -22.3578m)
	Women	-1,666.57m (-2,740.57m to -1,023.28m)	-778.449m (-1,397.38m to -465.179m)	-436.824m (-755.11m to -266.633m)	-208.198m (-439.178m to -112.788m)	-261.66m (-437.384m to -158.814m)	-124.891m (-226.715m to -74.0207m)	-65.3383m (-116.259m to -37.8499m)	-32.2814m (-61.5559m to -16.6856m)
Hypertension medical costs	Men	-969.971m (-1,321.82m to -696.915m)	-179.042m (-296.755m to -87.2995m)	-290.542m (-417.452m to -205.01m)	-63.145m (-106.131m to -28.9093m)	-197.093m (-271.818m to -140.575m)	-38.5978m (-61.0744m to -20.3556m)	-61.5264m (-86.9187m to -42.7291m)	-15.5811m (-25.2685m to -8.27631m)
	Women	-587.001m (-835.081m to -369.544m)	-225.074m (-326.525m to -145.468m)	-157.204m (-229.253m to -99.8575m)	-65.3715m (-96.2129m to -42.178m)	-92.1071m (-133.638m to -57.0408m)	-36.9331m (-54.1168m to -23.2286m)	-23.1777m (-34.0893m to -14.7901m)	-10.2317m (-15.0542m to -6.49733m)
Hypertension productivity costs	Men	-820.918m (-1,128.33m to -569.039m)	-235.655m (-354.351m to -144.401m)	-299.054m (-422.955m to -198.367m)	-91.4005m (-144.549m to -52.3003m)	-165.394m (-226.331m to -113.658m)	-48.6453m (-73.1298m to -29.8311m)	-62.0811m (-87.2646m to -40.3663m)	-20.4384m (-31.0779m to -11.8295m)
	Women	-238.169m (-328.618m to -156.688m)	-101.705m (-142.122m to -67.6037m)	-78.1912m (-112.637m to -51.4981m)	-35.7401m (-51.5852m to -23.6217m)	-37.3532m (-52.8429m to -23.1497m)	-16.5508m (-23.2881m to -10.5285m)	-11.4054m (-16.3916m to -7.03964m)	-5.43789m (-7.79943m to -3.35437m)
CHD medical costs	Men	-342.952m (-778.688m to -112.222m)	-95.6019m (-227.228m to -6.72173m)	-59.1899m (-155.625m to -13.7209m)	-11.6714m (-46.5621m to 10.7473m)	-72.1837m (-168.132m to -25.5795m)	-20.8196m (-51.856m to -3.33087m)	-14.765m (-38.2077m to -3.67803m)	-4.15547m (-12.7674m to 0.642826m)
	Women	-250.684m (-598.285m to -81.6699m)	-134.582m (-342.695m to -35.6784m)	-44.0296m (-126.98m to -8.87712m)	-24.4206m (-69.1058m to -0.864415m)	-38.4307m (-96.9309m to -12.6618m)	-21.5756m (-51.9005m to -5.39778m)	-7.74826m (-20.857m to -1.28198m)	-4.36665m (-11.9305m to -0.17757m)
CHD mortality productivity costs	Men	-381.167m (-1,732.02m to -61.2619m)	-121.216m (-653.197m to 133.674m)	-97.9025m (-711.416m to 38.0306m)	-23.8974m (-304.05m to 178.754m)	-79.0431m (-395.762m to -11.6372m)	-28.472m (-155.417m to 27.4974m)	-19.4786m (-112.256m to 4.26704m)	-4.92181m (-40.8226m to 31.7107m)

Model outputs	Sex	**Food System - Ever**	**Food System - Ever**	**Food System - Current**	**Food System - Current**	**Processed Food - Ever**	**Processed Food - Ever**	**Processed Food - Current**	**Processed Food - Current**
		Long-term compliance	Short-term compliance	Long-term compliance	Short-term compliance	Long-term compliance	Short-term compliance	Long-term compliance	Short-term compliance
	Women	-75.3255m (-455.64m to -0.462779m)	-37.915m (-311.822m to 20.7076m)	-29.7089m (-247.971m to 0.945874m)	-5.25237m (-165.783m to 20.5948m)	-11.3293m (-73.9871m to 0.50907m)	-5.32555m (-44.0885m to 3.87919m)	-1.63147m (-23.4472m to 0.608049m)	-0.351214m (-18.8828m to 1.6235m)
CHD morbidity productivity costs	Men	-86.565m (-231.161m to -22.9061m)	-30.5252m (-81.8729m to -5.69703m)	-30.7234m (-85.4018m to -7.11623m)	-8.39569m (-27.9788m to 2.56558m)	-16.1571m (-43.7919m to -4.35791m)	-5.87377m (-16.0723m to -1.01302m)	-6.87322m (-19.1631m to -1.62883m)	-2.20476m (-6.8522m to -0.0750617m)
	Women	-36.0412m (-106.56m to -9.46882m)	-20.6722m (-59.7477m to -4.29276m)	-13.8968m (-40.8969m to -2.40513m)	-7.61057m (-24.3605m to -0.484882m)	-5.51136m (-15.7827m to -1.21205m)	-3.22477m (-8.99249m to -0.441429m)	-2.08944m (-6.29998m to -0.370936m)	-1.18087m (-3.75538m to -0.0733372m)
CHD informal care costs	Men	-85.6695m (-218.34m to -26.9893m)	-26.8531m (-67.8253m to -4.43838m)	-19.4939m (-55.4631m to -4.8448m)	-4.89996m (-17.3415m to 1.91621m)	-17.6091m (-43.4845m to -5.77151m)	-5.52569m (-14.7617m to -1.32651m)	-4.62408m (-13.1447m to -1.17589m)	-1.43794m (-4.5145m to -0.0693873m)
	Women	-44.2675m (-115.926m to -13.6776m)	-24.4506m (-62.9828m to -6.95038m)	-9.60432m (-29.4949m to -2.08372m)	-5.53639m (-17.2806m to -0.510109m)	-6.73797m (-17.9844m to -2.15843m)	-3.92696m (-9.87411m to -1.04321m)	-1.62821m (-4.77153m to -0.335305m)	-0.933298m (-2.87853m to -0.0655785m)
Stroke medical costs	Men	-300.47m (-682.184m to -101.594m)	-118.38m (-263m to -26.2533m)	-44.3297m (-113.251m to -10.9051m)	-14.8592m (-48.1614m to 3.94898m)	-62.7945m (-142.913m to -22.7593m)	-24.4731m (-59.5776m to -6.99419m)	-11.3185m (-26.3366m to -3.27336m)	-4.52669m (-12.3369m to -0.16747m)
	Women	-174.558m (-414.04m to -56.4475m)	-95.5553m (-231.964m to -26.5575m)	-27.8217m (-73.3769m to -4.73705m)	-15.0433m (-43.5073m to -0.850259m)	-27.4082m (-66.4745m to -8.31408m)	-15.3852m (-39.6161m to -3.95795m)	-5.00346m (-13.8152m to -1.21715m)	-2.76829m (-8.54743m to -0.0833784m)
Stroke mortality productivity costs	Men	-170.599m (-781.118m to 9.14522m)	-63.9456m (-435.835m to 123.789m)	-40.8495m (-224.746m to 45.1818m)	-10.9305m (-126.752m to 98.335m)	-38.3728m (-181.075m to -1.41187m)	-14.6516m (-92.6281m to 19.2938m)	-8.13993m (-45.2846m to 6.50864m)	-2.47377m (-26.0123m to 16.6235m)
	Women	-55.8029m (-264.837m to 4.06367m)	-29.4831m (-184.603m to 27.4957m)	-12.3064m (-124.791m to 7.05384m)	-2.35517m (-75.7783m to 11.7053m)	-8.80112m (-40.0981m to 2.94983m)	-4.63368m (-30.616m to 5.6814m)	-0.477224m (-16.4855m to 1.28224m)	0m (-12.1993m to 2.83472m)
Stroke morbidity productivity costs	Men	-50.1946m (-127.212m to -16.2979m)	-20.1817m (-52.3848m to -3.81067m)	-17.3474m (-45.5229m to -3.79446m)	-5.37682m (-18.4184m to 1.82461m)	-9.67473m (-23.0063m to -3.04868m)	-3.94239m (-9.95849m to -0.642911m)	-3.97403m (-9.77384m to -1.15071m)	-1.46032m (-4.17152m to 0.0180707m)
	Women	-25.7784m (-70.3446m to -6.74734m)	-14.1817m (-41.064m to -2.69398m)	-10.0653m (-29.1148m to -2.02538m)	-5.36444m (-16.995m to -0.554859m)	-3.77826m (-10.4671m to -0.955518m)	-2.11007m (-6.31247m to -0.318554m)	-1.458m (-4.34046m to -0.232248m)	-0.776231m (-2.60121m to -0.00850394m)
Stroke informal care costs	Men	-166.925m (-474.287m to -48.0594m)	-55.4592m (-165.289m to -11.5871m)	-33.403m (-110.23m to -6.74346m)	-9.63413m (-39.6522m to 4.65071m)	-34.5101m (-102.846m to -9.68675m)	-11.5807m (-35.5m to -2.10365m)	-8.17084m (-25.2288m to -2.12775m)	-2.85274m (-10.2515m to 0.187334m)
	Women	-103.001m (-298.658m to -24.9896m)	-55.5819m (-167.693m to -11.5536m)	-22.5142m (-72.2946m to -3.4636m)	-12.0616m (-41.2025m to -1.26422m)	-14.8942m (-46.3864m to -3.82469m)	-8.41042m (-25.4203m to -1.77911m)	-3.45347m (-11.1869m to -0.751556m)	-1.85163m (-6.7542m to -0.152283m)
Change in policy costs:	Men	8,387.71m (3,069.95m	3,720.64m (1,394.25m to 7,864.1m)	8,162.99m (2,984.01m to 17,407.3m)	3,612.93m (1,352.55m to 7,640.49m)	9,679m (3,539.35m to 20,633.2m)	4,299.43m (1,611.13m to 9,097.74m)	9,916.31m (3,624.48m to 21,168.3m)	4,391.28m (1,643.21m to 9,292.79m)

Model outputs	Sex	**Food System - Ever**	**Food System - Ever**	**Food System - Current**	**Food System - Current**	**Processed Food - Ever**	**Processed Food - Ever**	**Processed Food - Current**	**Processed Food - Current**
		Long-term compliance	Short-term compliance	Long-term compliance	Short-term compliance	Long-term compliance	Short-term compliance	Long-term compliance	Short-term compliance
		to 17,869.7m)							
	Women	8,234.05m (3,007.05m to 17,527.4m)	3,638.67m (1,360.32m to 7,685.4m)	8,461.82m (3,092.5m to 17,989.8m)	3,747.1m (1,403.29m to 7,909.01m)	6,936.54m (2,535.79m to 14,769.4m)	3,056.49m (1,144.3m to 6,453.85m)	6,701.94m (2,450.54m to 14,234.3m)	2,965.15m (1,111.8m to 6,258.8m)
Policy admin costs	Men	81.8897m (59.0139m to 111.115m)	81.889m (59.013m to 111.113m)	79.7245m (57.5375m to 108.385m)	79.7256m (57.536m to 108.384m)	94.4956m (68.2264m to 128.464m)	94.496m (68.2267m to 128.464m)	96.9484m (69.9195m to 131.764m)	96.9498m (69.9192m to 131.765m)
	Women	80.4071m (58.1327m to 109.41m)	80.4074m (58.1331m to 109.41m)	82.6655m (59.6371m to 112.194m)	82.6655m (59.6386m to 112.195m)	67.7108m (48.9227m to 92.1254m)	67.7121m (48.9224m to 92.1255m)	65.5038m (47.2296m to 88.8253m)	65.5051m (47.2299m to 88.8237m)
Policy monitoring costs	Men	14.4333m (10.5601m to 19.4706m)	14.4331m (10.5601m to 19.4707m)	14.0799m (10.3131m to 19.0177m)	14.0798m (10.3128m to 19.0177m)	16.6495m (12.2131m to 22.4593m)	16.6494m (12.213m to 22.4589m)	17.1222m (12.534m to 23.11m)	17.1222m (12.5339m to 23.1099m)
	Women	14.2334m (10.4403m to 19.2271m)	14.2336m (10.4404m to 19.227m)	14.5861m (10.696m to 19.6718m)	14.5864m (10.6961m to 19.6719m)	12.0309m (8.81362m to 16.2379m)	12.031m (8.81369m to 16.2377m)	11.566m (8.4626m to 15.5887m)	11.5662m (8.46251m to 15.5889m)
Policy industry costs	Men	8,300.31m (2,974.67m to 17,782.7m)	3,628.95m (1,302m to 7,777.15m)	8,074.81m (2,895.51m to 17,322.5m)	3,521.41m (1,263.81m to 7,555.69m)	9,563.89m (3,429.92m to 20,533.2m)	4,191.89m (1,503.72m to 8,997.77m)	9,813.62m (3,517.25m to 21,065.5m)	4,280.96m (1,535.44m to 9,190.03m)
	Women	8,138.93m (2,917.31m to 17,442m)	3,543m (1,270.57m to 7,599.98m)	8,369.91m (3,000.83m to 17,902.2m)	3,654.12m (1,311.33m to 7,821.44m)	6,862.56m (2,460.49m to 14,697.6m)	2,977.8m (1,068.61m to 6,382m)	6,625.46m (2,378.07m to 14,165.3m)	2,891.54m (1,039.26m to 6,189.75m)
Total net cost (medical perspective)	Men	-1,637.53m (-2,491.32m to -1,101.07m)	-396.304m (-674.428m to -210.377m)	-399.096m (-603.705m to -270.48m)	-91.297m (-162.223m to -43.1094m)	-338.071m (-518.239m to -222.839m)	-85.525m (-139.304m to -48.9195m)	-89.8581m (-134.419m to -59.4815m)	-24.9141m (-40.1351m to -13.4623m)
	Women	-1,034.07m (-1,637.29m to -618.706m)	-460.816m (-770.909m to -272.808m)	-234.697m (-365.437m to -142.887m)	-108.569m (-171.325m to -62.9799m)	-161.122m (-267.948m to -95.7661m)	-75.435m (-126.261m to -43.7854m)	-37.1439m (-61.5488m to -21.8229m)	-17.867m (-30.2337m to -9.77065m)
Total net cost (societal perspective)	Men	4,719.32m (-1,225.7m to 14,275.9m)	2,712.91m (250.57m to 6,919.4m)	7,111.16m (1,821.79m to 16,189.3m)	3,352.67m (1,078.19m to 7,401.97m)	8,954.09m (2,662.49m to 19,815m)	4,077.1m (1,356.43m to 8,890.77m)	9,699.27m (3,364.85m to 20,918.1m)	4,321.19m (1,587.71m to 9,257.41m)
	Women	6,545.46m (1,183.36m to 15,497.6m)	2,840.79m (449.551m to 6,769.26m)	8,040.01m (2,599.85m to 17,451.1m)	3,540.6m (1,163.18m to 7,617.39m)	6,683.82m (2,225.18m to 14,416.2m)	2,928.47m (1,014.87m to 6,254.21m)	6,640.32m (2,385.89m to 14,145.5m)	2,937.49m (1,079.59m to 6,213.38m)

Model outputs	Sex	**Food System - Ever**	**Food System - Ever**	**Food System - Current**	**Food System - Current**	**Processed Food - Ever**	**Processed Food - Ever**	**Processed Food - Current**	**Processed Food - Current**
		Long-term compliance	Short-term compliance	Long-term compliance	Short-term compliance	Long-term compliance	Short-term compliance	Long-term compliance	Short-term compliance
Net monetary benefit in billion (valuing QALYs at $100,000)	Men	6,478.33m (-3,079.9m to 13,375.9m)	493.435m (-4,019.86m to 3,414.58m)	-2,794.19m (-12,139m to 2,642.17m)	-2,036.09m (-6,002.4m to 317.432m)	-6,717.41m (-17,775.7m to -445.556m)	-3,421.51m (-8,281.03m to -704.35m)	-8,862.19m (-20,113.3m to -2,518.15m)	-4,054.37m (-8,993.71m to -1,273.19m)
	Women	218.626m (-9,249.88m to 5,795.57m)	209.813m (-3,856.24m to 2,709.85m)	-5,548.73m (-15,032.9m to -200.558m)	-2,372.51m (-6,570.75m to 10.5041m)	-5,686.77m (-13,502.2m to -1,200.57m)	-2,467.04m (-5,832.23m to -514.346m)	-6,311.09m (-13,825.3m to -2,055.53m)	-2,783.51m (-6,057.98m to -918.953m)
Incremental cost-effectiveness ratio (2017 US Dollars per QALY)	Men	41,623.7 (-9,719.19 to 131,427)	84,447.2 (7,474.44 to 264,558)	164,715 (40,987.9 to 382,614)	253,830 (79,185.4 to 649,116)	399,306 (120,947 to 933,123)	639,242 (210,651 to 1,514,060)	1,145,100 (401,346 to 2,595,320)	1,648,990 (551,059 to 3,950,370)
	Women	96,589.8 (18,313.6 to 256,214)	92,445.2 (16,311.7 to 242,213)	331,385 (107,896 to 770,788)	304,502 (99,132.5 to 720,291)	664,392 (219,930 to 1,517,970)	625,883 (212,514 to 1,438,210)	2,076,520 (758,174 to 4,733,160)	1,910,840 (655,564 to 4,287,300)

Table S 5. Total policy costs (discounted) over ten years, in 2017 million US dollars (95% uncertainty intervals in brackets).

	**Short term compliance scenario ($million)**	**Long term compliance scenario ($million)**
Total Industry costs	7,146<br>(3,153; 13,393)	16,406<br>(11,882; 30,747)
Determine response to regulation	766<br>(316; 1,483)	1,759<br>(725; 3,405)
Project management	2,065<br>(864; 3,965)	4,741<br>(1,984; 9,103)
Product reformulation/process modification	2,580<br>(1,174; 4,747)	5,922<br>(2,695; 10,897)
Packaging assessment	329<br>(144; 629)	756<br>(332; 1,443)
Production scale-up testing	1,406<br>(655; 2,569)	3,228<br>(1,045; 5,898)
Government Administrative cost	164<br>(131; 196)	164<br>(131; 196)
Government Monitoring & Evaluation	29<br>(23; 35)	29<br>(23; 35)
Total costs	7,339<br>(3,307; 13,624)	16,599<br>(12,037; 30,978)

Table S 6. Cost effectiveness model results for 20 years from 2017-2036– short term compliance scenario.

	Food System – Ever Workers	Food System – Current Workers	Processed Food Industry – Ever Workers	Processed Food Industry – Current Workers
**Population modelled**	19.0m (18.7m to 19.2m)	7.3m (6.7m to 8.0m)	3.1m (3.0m to 3.1m)	1.1m (1.0m to 1.2m)
**Median sodium consumption in 2036 (mg/d)**	2,776 (2,766 to 2,786)	2,927 (2,910 to 2,944)	2,819 (2,807 to 2,829)	2,968 (2,947 to 2,986)
**Median SBP in 2036 (mmHg)**	115.1 (114.9 to 115.3)	114.1 (113.9 to 114.3)	115.9 (115.7 to 116.1)	115.5 (115.3 to 115.7)
**CVD cases prevented or postponed (undiscounted)**	10,400 (5,197.5 to 17,100)	2,600 (500 to 5,300)	1,880 (920 to 3,182)	540 (140 to 1,080)
**CVD deaths prevented or postponed (undiscounted)**	1,100 (100 to 2,400)	500 (0 to 1,200)	220 (0 to 480)	80 (0 to 200)
**Non-CVD deaths prevented or postponed (undiscounted)**	600 (0 to 1,700)	300 (0 to 1,000)	100 (0 to 320)	40 (0 to 160)
**QALYs gained (discounted)**	62,447 (49,840 to 76,700)	24,666 (18,321 to 32,492)	11,106 (8,834 to 13,716)	4,245 (3,139 to 5,517)
**Change in health-related costs: (discounted)**	-1.8bn (-3.1bn to -1.1bn)	-0.468bn (-0.959bn to -0.224bn)	-0.338bn (-0.587bn to -0.217bn)	-0.096bn (-0.165bn to -0.053bn)
**Change in policy and industry costs: (discounted)**	7.4bn (2.8bn to 15.5bn)	7.4bn (2.8bn to 15.5bn)	7.4bn (2.8bn to 15.5bn)	7.4bn (2.8bn to 15.5bn)
**Total net cost (societal perspective) (discounted)**	5.5bn (0.701bn to 13.6bn)	6.8bn (2.2bn to 14.8bn)	7.0bn (2.4bn to 15.1bn)	7.3bn (2.7bn to 15.5bn)
**Net monetary benefit (valuing QALYs at $100,000) (discounted)**	0.681bn (-7.3bn to 5.8bn)	-4.4bn (-12.5bn to 0.262bn)	-5.9bn (-14.2bn to -1.2bn)	-6.8bn (-15.1bn to -2.2bn)
**Incremental cost-effectiveness ratio (2017 US Dollars per QALY) (discounted)**	88,897 (11,688 to 227,790)	277,827 (88,159 to 628,445)	631,380 (208,041 to 1,439,560)	1,715,790 (617,857 to 3,902,420)
**Incremental cost-effectiveness ratio (2017 US Dollars per QALY) (discounted) - Men**	84,447.2 (7,474.44 to 264,558)	253,830 (79,185.4 to 649,116)	639,242 (210,651 to 1,514,060)	1,648,990 (551,059 to 3,950,370)
**Incremental cost-effectiveness ratio (2017 US Dollars per QALY) (discounted) - Women**	92,445.2 (16,311.7 to 242,213)	304,502 (99,132.5 to 720,291)	625,883 (212,514 to 1,438,210)	1,910,840 (655,564 to 4,287,300)

Table S 6. Full Impact inventory (95% Uncertainty Intervals in brackets)

Model outputs	Food System - Ever<br>**Long-term compliance**	**Short-term compliance**	Food System - Ever<br>**Long-term compliance**	**Short-term compliance**	Food System - Current<br>**Long-term compliance**	**Short-term compliance**	Food System - Current<br>**Long-term compliance**	**Short-term compliance**
Population	18,952,200 (18,677,700 to 19,198,000)	18,948,600 (18,672,600 to 19,193,600)	7,349,250 (6,661,030 to 8,003,300)	7,347,250 (6,659,130 to 8,002,510)	3,091,140 (3,039,020 to 3,136,070)	3,090,600 (3,038,210 to 3,135,220)	1,116,060 (1,017,320 to 1,213,520)	1,115,740 (1,017,120 to 1,213,190)
Median sodium consumption in 2036 (mg/d)	2,212.97 (2,204.09 to 2,221.01)	2,775.85 (2,766 to 2,786.32)	2,326.88 (2,311.31 to 2,340.6)	2,927.46 (2,910.26 to 2,944.31)	2,242.66 (2,234.34 to 2,251.38)	2,818.64 (2,806.9 to 2,829.39)	2,358.57 (2,343.11 to 2,374.88)	2,967.58 (2,946.65 to 2,985.91)
Median SBP in 2036 (mmHg)	114 (113.9 to 114.2)	115.1 (114.9 to 115.3)	113.2 (112.9 to 113.4)	114.1 (113.9 to 114.3)	114.8 (114.6 to 115)	115.9 (115.7 to 116.1)	114.4 (114.2 to 114.7)	115.5 (115.3 to 115.7)
CVD cases prevented or postponed	38,700 (21,795 to 65,105)	10,400 (5,197.5 to 17,100)	10,100 (4,700 to 19,400)	2,600 (500 to 5,300)	7,140 (3,899 to 12,062)	1,880 (920 to 3,182)	2,020 (960 to 3,742)	540 (140 to 1,080)
CVD deaths prevented or postponed	3,000 (1,300 to 5,800)	1,100 (100 to 2,400)	1,200 (300 to 2,500)	500 (0 to 1,200)	600 (260 to 1,101)	220 (0 to 480)	200 (60 to 421)	80 (0 to 200)
Non-CVD deaths prevented or postponed	3,500 (1,900 to 5,700)	600 (0 to 1,700)	1,400 (600 to 2,500)	300 (0 to 1,000)	660 (300 to 1,041)	100 (0 to 320)	220 (60 to 400)	40 (0 to 160)
Life years gained	43,300 (25,592.5 to 63,402.5)	15,000 (3,100 to 28,007.5)	17,800 (8,797.5 to 28,602.5)	6,400 (1,097.5 to 14,400)	8,240 (4,940 to 12,482)	2,840 (699 to 5,520)	3,020 (1,460 to 4,981)	1,020 (40 to 2,280)
Discounted QALYs gained	180,535 (150,159 to 209,477)	62,447 (49,840 to 76,700)	67,411 (54,923 to 80,639)	24,666 (18,321 to 32,492)	32,364 (27,114 to 37,415)	11,106 (8,834 to 13,716)	11,581 (9,674 to 13,981)	4,245 (3,139 to 5,517)
Change in health-related costs:	-5,196.62m (-8,344.2m to -3,498.71m)	-1,771.83m (-3,078.96m to -1,112.47m)	-1,419.84m (-2,404.33m to -970.805m)	-468.329m (-958.781m to -223.899m)	-988.709m (-1,603.71m to -658.024m)	-338.179m (-587.149m to -217.242m)	-277.371m (-448.494m to -188.625m)	-96.0074m (-164.754m to -52.9767m)
Hypertension medical costs	-1,555.06m (-2,132.71m to -1,083.47m)	-403.96m (-580.009m to -268.659m)	-446.663m (-629.222m to -315.507m)	-128.522m (-193.488m to -82.6783m)	-289.09m (-400.003m to -198.383m)	-75.6453m (-109.487m to -51.7428m)	-84.4498m (-118.7m to -58.6602m)	-25.8811m (-38.1537m to -17.2449m)
Hypertension productivity costs	-1,060.32m (-1,431.31m to -747.764m)	-337.616m (-476.285m to -227.038m)	-378.925m (-519.499m to -256.348m)	-127.642m (-189.043m to -79.4524m)	-202.756m (-275.448m to -140.21m)	-65.3791m (-93.5641m to -42.5702m)	-73.1963m (-101.777m to -49.3519m)	-25.793m (-37.502m to -16.17m)
CHD medical costs	-593.5m (-1,371.11m to -203.368m)	-236.395m (-517.746m to -64.9204m)	-103.013m (-274.925m to -27.8738m)	-36.6309m (-102.331m to -1.86754m)	-110.74m (-252.7m to -39.6261m)	-42.8369m (-96.9456m to -13.9185m)	-22.2899m (-57.1316m to -6.71338m)	-8.61247m (-22.4191m to -1.4749m)
CHD mortality productivity costs	-462.075m (-2,223.52m to -109.211m)	-163.404m (-873.132m to 89.1984m)	-130.452m (-939.986m to 13.2164m)	-39.0994m (-394.851m to 155.968m)	-91.5906m (-466.304m to -17.7497m)	-35.0144m (-181.315m to 20.5947m)	-23.6219m (-130.984m to 1.81719m)	-7.32264m (-47.5488m to 25.2381m)

Model outputs	Food System - Ever<br>**Long-term compliance**	**Short-term compliance**	Food System - Ever<br>**Long-term compliance**	**Short-term compliance**	Food System - Current<br>**Long-term compliance**	**Short-term compliance**	Food System - Current<br>**Long-term compliance**	**Short-term compliance**
CHD morbidity productivity costs	-122.918m (-328.367m to -35.8003m)	-51.3341m (-129.921m to -13.0352m)	-45.4094m (-124.99m to -11.4475m)	-16.3502m (-47.9419m to -1.20738m)	-21.9952m (-59.0545m to -6.32717m)	-9.1826m (-24.2121m to -2.2117m)	-9.04024m (-24.3279m to -2.34399m)	-3.47869m (-9.77902m to -0.50776m)
CHD informal care costs	-129.807m (-328.187m to -40.4427m)	-52.0417m (-125.061m to -14.4585m)	-29.2109m (-83.6978m to -8.17968m)	-10.8757m (-31.5558m to -0.934244m)	-24.3575m (-61.1369m to -8.87564m)	-9.61414m (-23.963m to -3.00446m)	-6.28613m (-17.4927m to -1.74132m)	-2.39022m (-7.11907m to -0.519744m)
Stroke medical costs	-476.796m (-1,066.01m to -169.342m)	-214.03m (-458.617m to -70.8785m)	-71.5327m (-178.354m to -21.0467m)	-31.1066m (-78.8471m to -3.76434m)	-91.126m (-204.993m to -36.0706m)	-40.579m (-94.0912m to -15.0868m)	-16.5065m (-38.5691m to -5.53694m)	-7.50741m (-18.3753m to -1.60372m)
Stroke mortality productivity costs	-233.867m (-1,021.45m to -19.8559m)	-98.1069m (-560.762m to 97.7585m)	-61.5339m (-335.994m to 30.9758m)	-23.7578m (-169.891m to 92.1473m)	-48.407m (-197.15m to -4.57282m)	-20.1097m (-107.414m to 15.1653m)	-10.4188m (-52.971m to 6.11532m)	-3.97578m (-34.758m to 13.9634m)
Stroke morbidity productivity costs	-76.4088m (-194.245m to -25.1501m)	-35.3491m (-89.8255m to -9.77656m)	-27.1203m (-71.9767m to -7.81076m)	-11.3708m (-30.8831m to -0.902907m)	-13.4578m (-32.5319m to -4.63387m)	-6.0738m (-14.9566m to -1.63939m)	-5.45771m (-13.7004m to -1.58089m)	-2.32953m (-5.98265m to -0.394071m)
Stroke informal care costs	-274.762m (-763.641m to -76.8388m)	-114.268m (-313.583m to -29.8611m)	-58.0496m (-176.455m to -13.8976m)	-22.3681m (-72.9778m to -1.70618m)	-49.6244m (-148.955m to -14.305m)	-20.2488m (-59.5946m to -5.71587m)	-11.711m (-35.595m to -3.37212m)	-4.86513m (-15.6216m to -0.827276m)
Change in policy costs:	16,624.8m (6,076.51m to 35,397.1m)	7,360.03m (2,755.84m to 15,549.5m)	16,624.8m (6,076.51m to 35,397.1m)	7,360.03m (2,755.84m to 15,549.5m)	16,619.6m (6,075.02m to 35,402.6m)	7,359.74m (2,755.44m to 15,551.6m)	16,619.6m (6,075.02m to 35,402.6m)	7,359.74m (2,755.44m to 15,551.6m)
Policy admin costs	162.391m (117.175m to 220.579m)	162.391m (117.175m to 220.579m)	162.391m (117.175m to 220.579m)	162.391m (117.175m to 220.579m)	162.474m (117.149m to 220.589m)	162.474m (117.149m to 220.589m)	162.474m (117.149m to 220.589m)	162.474m (117.149m to 220.589m)
Policy monitoring costs	28.6697m (21.0048m to 38.6977m)	28.6697m (21.0048m to 38.6977m)	28.6697m (21.0048m to 38.6977m)	28.6697m (21.0048m to 38.6977m)	28.6855m (21.0041m to 38.6988m)	28.6855m (21.0041m to 38.6988m)	28.6855m (21.0041m to 38.6988m)	28.6855m (21.0041m to 38.6988m)
Policy industry costs	16,446.3m (5,896.34m to 35,224.7m)	7,177.12m (2,575.14m to 15,377.1m)	16,446.3m (5,896.34m to 35,224.7m)	7,177.12m (2,575.14m to 15,377.1m)	16,444.4m (5,895.32m to 35,230.8m)	7,176.29m (2,574.7m to 15,379.8m)	16,444.4m (5,895.32m to 35,230.8m)	7,176.29m (2,574.7m to 15,379.8m)
Total net cost (medical perspective)	-2,676.52m (-4,122.04m to -1,732.59m)	-869.402m (-1,370.63m to -533.015m)	-636.398m (-950.69m to -421.015m)	-199.046m (-311.917m to -126.147m)	-500.438m (-770.048m to -323.369m)	-161.394m (-253.476m to -101.81m)	-126.561m (-195.135m to -82.4514m)	-42.4149m (-66.1704m to -26.5092m)
Total net cost (societal perspective)	11,211.7m (260.732m to 29,934.6m)	5,505.73m (700.908m to 13,581.1m)	15,124.4m (4,386.31m to 33,388.2m)	6,849.78m (2,213.9m to 14,795.9m)	15,641.1m (4,954.06m to 34,231.2m)	7,021.86m (2,400.57m to 15,145m)	16,353.1m (5,773.18m to 35,063.6m)	7,262.39m (2,676.62m to 15,472.3m)
Net monetary benefit in billion (valuing QALYs at $100,000)	6,798m (-12,056.1m to 18,370.9m)	681.259m (-7,297.79m to 5,760.18m)	-8,330.08m (-27,466m to 2,219.04m)	-4,360.54m (-12,470.5m to 262.379m)	-12,375m (-31,392m to -1,670.59m)	-5,868.13m (-14,199.9m to -1,225.73m)	-15,152.5m (-33,938.6m to -4,576.3m)	-6,848.68m (-15,078.8m to -2,192.61m)
Incremental cost-effectiveness ratio (2017 US Dollars per QALY)	62,058.4 (1,461.18 to 170,966)	88,897.3 (11,687.6 to 227,790)	223,601 (66,409 to 508,341)	277,827 (88,158.9 to 628,445)	485,539 (147,984 to 1,094,030)	631,380 (208,041 to 1,439,560)	1,403,840 (485,151 to 3,119,530)	1,715,790 (617,857 to 3,902,420)

Table S 7. Results by Age Group (30-64, 65-84)

Model outputs	Age group	Food System - Ever	Food System - Ever	Food System - Current	Food System - Current	Processed Food - Ever	Processed Food - Ever	Processed Food -Current	Processed Food -Current
		Long-term compliance	Short-term compliance	Long-term compliance	Short-term compliance	Long-term compliance	Short-term compliance	Long-term compliance	Short-term compliance
Population	30-64	12,728,000 (12,607,600 to 12,839,800)	12,727,600 (12,606,200 to 12,838,800)	6,690,950 (6,062,510 to 7,286,800)	6,690,200 (6,061,920 to 7,286,700)	1,971,340 (1,948,410 to 1,992,140)	1,971,240 (1,948,140 to 1,991,890)	969,660 (879,133 to 1,058,030)	969,620 (879,075 to 1,057,950)
	65-84	6,220,300 (6,050,460 to 6,371,110)	6,217,000 (6,046,690 to 6,367,810)	655,600 (592,678 to 718,930)	654,150 (591,385 to 717,632)	1,120,060 (1,088,320 to 1,147,590)	1,119,540 (1,087,750 to 1,146,920)	146,480 (136,899 to 155,921)	146,300 (136,739 to 155,722)
Median sodium consumption in 2036 (mg/d)	30-64	2,323.62 (2,310.95 to 2,335.27)	2,929.09 (2,913.16 to 2,943.3)	2,353.88 (2,337.24 to 2,369.46)	2,963.02 (2,943.89 to 2,981.66)	2,376.44 (2,362.47 to 2,390.44)	2,993.25 (2,975.06 to 3,009.72)	2,413.91 (2,396.99 to 2,429.68)	3,038.71 (3,015.99 to 3,058.67)
	65-84	2,001.78 (1,986.97 to 2,015.79)	2,505.74 (2,487.86 to 2,522.57)	2,126.86 (2,101.2 to 2,149.7)	2,639.92 (2,608.8 to 2,670.45)	2,048.22 (2,033.5 to 2,063.06)	2,565.92 (2,549.16 to 2,581.8)	2,069.85 (2,035.92 to 2,097.65)	2,581.48 (2,545.62 to 2,613.65)
Median SBP in 2036 (mmHg)	30-64	113.2 (113 to 113.4)	114.2 (114 to 114.3)	112.9 (112.7 to 113.1)	113.8 (113.6 to 114)	113.9 (113.7 to 114.1)	115 (114.8 to 115.1)	114.2 (114 to 114.4)	115.2 (115 to 115.4)
	65-84	116.3 (115.9 to 116.6)	117.6 (117.2 to 117.9)	116.1 (115.5 to 116.7)	117.4 (116.8 to 118)	116.6 (116.3 to 117)	117.9 (117.6 to 118.3)	116.5 (116 to 117.1)	117.9 (117.3 to 118.4)
CVD cases prevented or postponed	30-64	21,050 (10,497.5 to 38,915)	6,900 (3,200 to 12,202.5)	8,200 (3,600 to 16,202.5)	2,300 (597.5 to 5,000)	3,720 (1,820 to 6,922)	1,220 (559 to 2,220)	1,580 (740 to 3,121)	480 (120 to 1,000)
	65-84	17,500 (10,400 to 26,817.5)	3,300 (597.5 to 6,500)	1,800 (700 to 3,600)	300 (-600 to 1,100)	3,460 (2,058 to 5,481)	640 (140 to 1,320)	400 (160 to 760)	60 (-100 to 260)
CVD deaths prevented or postponed	30-64	1,100 (300 to 2,400)	400 (0 to 1,200)	300 (0 to 900)	100 (0 to 500)	200 (59 to 460)	80 (0 to 240)	40 (0 to 140)	0 (0 to 80)
	65-84	1,800 (700 to 3,600)	600 (0 to 1,600)	900 (200 to 1,900)	400 (0 to 1,000)	380 (139 to 720)	140 (0 to 320)	160 (40 to 340)	60 (0 to 180)
Non-CVD deaths prevented or postponed	30-64	1,500 (600 to 2,700)	500 (0 to 1,200)	500 (0 to 1,100)	100 (0 to 500)	280 (100 to 500)	80 (0 to 220)	60 (0 to 160)	0 (0 to 80)
	65-84	2,000 (800 to 3,502.5)	100 (0 to 1,000)	900 (300 to 1,800)	200 (0 to 800)	380 (140 to 660)	20 (0 to 200)	140 (20 to 320)	40 (0 to 120)

Life years gained	30-64	25,500 (14,297.5 to 38,402.5)	8,200 (797.5 to 16,505)	11,900 (5,497.5 to 20,000)	4,700 (800 to 10,102.5)	5,080 (2,680 to 7,940)	1,660 (139 to 3,521)	2,100 (980 to 3,620)	760 (80 to 1,701)
	65-84	13,600 (6,400 to 22,502.5)	5,600 (0 to 12,502.5)	3,800 (400 to 8,802.5)	1,100 (0 to 5,002.5)	2,380 (1,079 to 4,103)	1,000 (0 to 2,201)	540 (0 to 1,302)	120 (0 to 681)
Discounted QALYs gained (million)	30-64	0.122691m (0.100727m to 0.144799m)	0.0481288m (0.0373432 m to 0.0605566m)	0.0555789m (0.0451521m to 0.0669165m)	0.0209612m (0.0152952m to 0.027348m)	0.0211292m (0.0178236m to 0.0249173m)	0.0082916m (0.00642252m to 0.0105581m)	0.00937865m (0.00772316m to 0.0114413m)	0.00356327m (0.00257293m to 0.00465756m)
	65-84	0.0578723m (0.0445638m to 0.0701495m)	0.014477m (0.00938346 m to 0.0200992m)	0.0117744m (0.00794478m to 0.0164338m)	0.00365273m (0.0014404m to 0.00676102m)	0.0111131m (0.00864247m to 0.0135081m)	0.00282326m (0.00175814m to 0.0039601m)	0.0021702m (0.00148702m to 0.00307487m)	0.000662421m (0.000268812m to 0.00118134m)
Change in health-related costs:	30-64	-2,901.84m (-4,945.07m to -1,960.44m)	-1,128.1m (-2,084.98m to -681.168m)	-1,111.87m (-1,804.58m to -769.071m)	-374.516m (-720.73m to -133.666m)	-529.911m (-985.244m to -356.547m)	-209.417m (-383.253m to -129.262m)	-210.375m (-335.325m to -142.54m)	-75.223m (-124.443m to -33.4854m)
	65-84	-2,289.44m (-3,539.58m to -1,457.43m)	-647.303m (-1,178.46m to -313.089m)	-301.855m (-680.632m to -157.354m)	-86.2939m (-318.141m to 2.1012m)	-456.347m (-713.861m to -291.314m)	-129.885m (-233.494m to -67.5033m)	-66.1335m (-123.662m to -34.6142m)	-20.3752m (-55.7079m to -3.06389m)
Hypertension medical costs	30-64	-863.967m (-1,189.82m to -596.91m)	-296.717m (-426.554m to -197.032m)	-373.198m (-525.283m to -262.282m)	-121.339m (-180.217m to -77.2451m)	-154.048m (-214.101m to -107.858m)	-53.4185m (-77.5613m to -35.4286m)	-69.3834m (-98.0468m to -48.0635m)	-23.5575m (-34.8776m to -15.3231m)
	65-84	-697.273m (-968.611m to -471.567m)	-108.648m (-175.589m to -54.5961m)	-73.7036m (-106.409m to -49.9214m)	-7.82397m (-18.8305m to 0.922843m)	-134.827m (-189.804m to -90.2823m)	-22.0343m (-35.776m to -11.8063m)	-15.2209m (-21.8499m to -9.95756m)	-2.28111m (-4.48234m to -0.431198m)
Hypertension productivity costs	30-64	-778.614m (-1,067.45m to -537.729m)	-288.35m (-415.457m to -190.773m)	-347.31m (-478.351m to -234.539m)	-124.069m (-183.994m to -78.0921m)	-144.781m (-199.041m to -99.0548m)	-54.8067m (-78.3073m to -35.2151m)	-66.5199m (-93.5264m to -44.2232m)	-24.6559m (-36.2397m to -15.3733m)
	65-84	-277.77m (-388.428m to -193.004m)	-47.957m (-77.4765m to -24.7186m)	-30.9409m (-43.7328m to -20.6135m)	-3.41304m (-8.14621m to 0.205704m)	-57.7824m (-79.1325m to -39.4082m)	-10.5175m (-16.9831m to -5.77368m)	-6.71919m (-9.49904m to -4.47725m)	-1.12045m (-2.12899m to -0.263152m)
CHD medical costs	30-64	-216.136m (-551.412m to -68.7348m)	-91.9195m (-226.558m to -21.3274m)	-78.2193m (-208.824m to -20.8943m)	-28.2018m (-82.2106m to -3.33522m)	-37.9945m (-94.1838m to -11.3319m)	-16.3242m (-40.0819m to -3.95249m)	-15.7959m (-42.708m to -4.19135m)	-6.12942m (-16.5642m to -0.877538m)
	65-84	-375.393m (-857.896m to -129.312m)	-142.65m (-309.491m to -31.463m)	-25.526m (-73.1034m to 1.85663m)	-7.70136m (-31.3689m to 12.1089m)	-71.9627m (-161.252m to -27.4582m)	-26.3987m (-60.7133m to -7.3089m)	-6.75706m (-17.8253m to -0.744924m)	-2.41603m (-8.14705m to 1.35013m)

CHD mortality productivity costs	30-64	-275.251m (-1,574.27m to -9.49366m)	-110.095m (-717.183m to 107.689m)	-51.5262m (-618.452m to 73.4556m)	-2.96173m (-251.225m to 199.661m)	-55.871m (-388.636m to 0m)	-23.6187m (-132.119m to 22.3355m)	-7.17234m (-91.6939m to 14.2414m)	0m (-37.0373m to 36.5596m)
	65-84	-176.095m (-647.727m to -14.5083m)	-51.0943m (-322.31m to 80.5661m)	-76.9661m (-359.033m to 2.12772m)	-29.7021m (-194.366m to 26.5966m)	-37.2513m (-115.522m to -4.28724m)	-11.7492m (-59.2487m to 16.9166m)	-15.4794m (-52.0174m to 0.276776m)	-5.94103m (-31.1488m to 5.09573m)
CHD morbidity productivity costs	30-64	-115.181m (-312.684m to -33.2103m)	-48.5351m (-123.249m to -12.0624m)	-44.0954m (-122.131m to -10.8808m)	-16.0112m (-46.7823m to -1.15911m)	-20.6022m (-55.9808m to -5.93339m)	-8.62736m (-22.612m to -2.01431m)	-8.76025m (-23.7139m to -2.20684m)	-3.39131m (-9.49181m to -0.50686m)
	65-84	-7.15595m (-17.5603m to -2.12841m)	-2.79464m (-7.56829m to -0.373118m)	-1.32896m (-3.81659m to -0.0219859m)	-0.371995m (-1.68288m to 0.716654m)	-1.30846m (-3.19904m to -0.407831m)	-0.524714m (-1.38678m to -0.0698275m)	-0.287703m (-0.857973m to -0.0353565m)	-0.0860081m (-0.363509m to 0.0971594m)
CHD informal care costs	30-64	-67.1932m (-186.834m to -19.2808m)	-28.4448m (-75.6592m to -7.0665m)	-24.7868m (-71.64m to -6.50016m)	-9.38425m (-28.4039m to -0.926789m)	-11.8639m (-32.249m to -3.53779m)	-5.0933m (-14.0253m to -1.31982m)	-5.09327m (-14.5768m to -1.34505m)	-1.99993m (-6.04266m to -0.3018m)
	65-84	-62.5022m (-145.17m to -22.7491m)	-22.9422m (-53.8856m to -4.99387m)	-4.44506m (-13.2112m to 0.395335m)	-1.21614m (-5.27169m to 1.99595m)	-12.4101m (-28.276m to -4.72384m)	-4.4908m (-10.338m to -1.17918m)	-1.15133m (-3.22126m to -0.133555m)	-0.401449m (-1.38243m to 0.235626m)
Stroke medical costs	30-64	-141.977m (-352.287m to -48.6219m)	-71.1667m (-167.329m to -20.4912m)	-48.858m (-123.602m to -14.8343m)	-21.6664m (-58.7502m to -3.57058m)	-24.95m (-59.007m to -8.81847m)	-12.4229m (-29.6644m to -3.72915m)	-9.94511m (-24.9911m to -3.09682m)	-4.72186m (-12.624m to -0.917622m)
	65-84	-333.046m (-742.712m to -110.598m)	-140.063m (-316.356m to -36.5633m)	-23.6625m (-61.1159m to -0.450491m)	-8.0405m (-28.7988m to 6.64973m)	-65.6613m (-147.716m to -24.7907m)	-27.8238m (-66.5617m to -8.28224m)	-6.2688m (-16.2182m to -1.15297m)	-2.48862m (-8.10231m to 0.872291m)
Stroke mortality productivity costs	30-64	-115.168m (-755.53m to 31.4251m)	-54.2325m (-412.831m to 109.66m)	-13.6894m (-178.339m to 72.4044m)	0m (-116.269m to 101.43m)	-21.9717m (-148.66m to 9.01147m)	-10.4766m (-99.1682m to 17.8091m)	-0.498311m (-32.3802m to 12.5345m)	0m (-19.9653m to 16.4282m)
	65-84	-111.079m (-387.011m to -0.742597m)	-42.9271m (-208.682m to 64.0302m)	-38.8767m (-172.652m to 9.58342m)	-19.0102m (-98.0333m to 25.8846m)	-25.6315m (-74.2868m to -0.524294m)	-10.0424m (-43.0854m to 12.4583m)	-7.24077m (-32.8048m to 2.63976m)	-3.34159m (-20.7837m to 6.30697m)
Stroke morbidity productivity costs	30-64	-71.5178m (-181.587m to -23.1739m)	-33.0728m (-84.9485m to -9.20257m)	-26.1798m (-69.8061m to -7.83396m)	-11.0435m (-30.6122m to -1.07009m)	-12.5479m (-30.3877m to -4.19158m)	-5.66607m (-14.3496m to -1.45241m)	-5.21475m (-13.2086m to -1.50041m)	-2.25576m (-5.83004m to -0.341793m)
	65-84	-4.99936m (-11.9981m to -1.50796m)	-2.12562m (-5.33779m to -0.251182m)	-0.921906m (-2.79157m to 0.0259412m)	-0.292654m (-1.38365m to 0.51303m)	-0.911936m (-2.28581m to -0.285156m)	-0.399871m (-1.00739m to -0.032656m)	-0.204787m (-0.603362m to 0m)	-0.0761333m (-0.300777m to 0.0750181m)
Stroke informal care costs	30-64	-122.482m (-373.041m to -30.9411m)	-57.4019m (-165.596m to -13.1999m)	-46.3075m (-145.477m to -10.485m)	-18.4457m (-62.0392m to -1.42637m)	-21.603m (-65.3634m to -6.01325m)	-9.70668m (-29.9298m to -2.01624m)	-8.97129m (-27.4726m to -2.27706m)	-3.85935m (-13.1106m to -0.583289m)

	65-84	-148.188m (-411.588m to -42.7429m)	-54.9587m (-155.397m to -10.6652m)	-11.2574m (-35.4521m to 0.311977m)	-3.48524m (-15.2891m to 4.78651m)	-28.2517m (-81.2523m to -7.96993m)	-10.3554m (-29.6171m to -1.88145m)	-2.74707m (-8.65518m to -0.399595m)	-1.01311m (-3.869m to 0.672934m)
Change in policy costs:	30-64	13,782.9m (5,037.69m to 29,405.3m)	6,355.56m (2,373.2m to 13,460.5m)	15,315.5m (5,598.42m to 32,606.9m)	6,789.62m (2,542.18m to 14,341.2m)	13,557m (4,946.97m to 28,865.9m)	6,300.86m (2,348.57m to 13,305.8m)	15,212.7m (5,561.41m to 32,409.3m)	6,758.48m (2,530.66m to 14,287.8m)
	65-84	2,838.37m (1,038.97m to 5,992.68m)	1,008.23m (382.654m to 2,099.59m)	1,308.45m (477.91m to 2,790.21m)	570.407m (212.705m to 1,208.27m)	3,062.61m (1,125.46m to 6,536.73m)	1,058.26m (402.404m to 2,242.19m)	1,407.07m (513.613m to 2,993.33m)	599.207m (224.019m to 1,263.8m)
Policy admin costs	30-64	132.653m (95.6471m to 179.963m)	132.654m (95.6467m to 179.964m)	149.541m (107.911m to 203.107m)	149.542m (107.91m to 203.108m)	130.034m (93.533m to 176.698m)	130.033m (93.5337m to 176.7m)	148.518m (107.088m to 201.704m)	148.515m (107.088m to 201.703m)
	65-84	29.82m (21.5275m to 40.5291m)	29.8194m (21.5279m to 40.5274m)	12.8375m (9.26408m to 17.4722m)	12.8348m (9.26486m to 17.4716m)	32.3799m (23.4273m to 43.8905m)	32.3766m (23.4267m to 43.8893m)	13.9278m (10.0613m to 18.8855m)	13.929m (10.0607m to 18.8859m)
Policy monitoring costs	30-64	23.0223m (16.8478m to 31.0252m)	23.0224m (16.848m to 31.0253m)	26.387m (19.3469m to 35.6213m)	26.3872m (19.3471m to 35.6213m)	22.4935m (16.4884m to 30.3577m)	22.4936m (16.4884m to 30.3579m)	26.1798m (19.1784m to 35.3128m)	26.1799m (19.1787m to 35.3129m)
	65-84	5.66863m (4.15343m to 7.6598m)	5.66842m (4.15336m to 7.65962m)	2.27951m (1.67184m to 3.07641m)	2.2793m (1.67159m to 3.07635m)	6.19052m (4.51177m to 8.33161m)	6.19019m (4.51173m to 8.33133m)	2.50209m (1.82984m to 3.36507m)	2.50204m (1.82985m to 3.36425m)
Policy industry costs	30-64	13,638.9m (4,890.83m to 29,264.7m)	6,205.52m (2,225.95m to 13,320m)	15,153.6m (5,432.51m to 32,448.2m)	6,621.7m (2,375.78m to 14,182.5m)	13,406.4m (4,803.78m to 28,728.9m)	6,146.96m (2,204.42m to 13,168.7m)	15,056.8m (5,397.15m to 32,252.2m)	6,593m (2,365.41m to 14,130.7m)
	65-84	2,802.63m (1,005.51m to 5,960.01m)	972.101m (349.193m to 2,063.9m)	1,293.07m (463.096m to 2,776.54m)	555.524m (198.848m to 1,194.59m)	3,022.87m (1,091.4m to 6,501.94m)	1,024.89m (368.954m to 2,211.03m)	1,391.44m (498.177m to 2,978.55m)	583.29m (209.288m to 1,249.03m)
Total net cost (medical perspective)	30-64	-1,239.93m (-1,834.41m to -839.103m)	-468.664m (-709.459m to -309.993m)	-511.081m (-755.225m to -338.607m)	-173.91m (-269.411m to -110.776m)	-222.089m (-330.332m to -147.054m)	-83.8296m (-126.082m to -54.7958m)	-97.6435m (-147.731m to -64.7084m)	-34.9981m (-53.7192m to -22.2799m)
	65-84	-1,429.41m (-2,285.97m to -887.187m)	-395.791m (-703.064m to -213.378m)	-125.753m (-207.669m to -71.724m)	-24.9468m (-57.4425m to 2.82437m)	-278.243m (-452.075m to -176.502m)	-78.1558m (-136.903m to -41.6984m)	-29.0089m (-49.5677m to -16.4448m)	-7.47227m (-16.2871m to -1.72159m)

Total net cost (societal perspective)	30-64	10,759m (1,621.45m to 26,069.3m)	5,173.56m (1,055.17m to 12,380.9m)	14,103.2m (4,361.56m to 31,033.1m)	6,393.57m (2,120.83m to 13,758.1m)	13,005.6m (4,321.25m to 28,243.6m)	6,074.06m (2,126.6m to 13,072.5m)	15,005.3m (5,359.03m to 32,166.9m)	6,678.54m (2,466.93m to 14,235.9m)
	65-84	551.689m (-1,660.76m to 3,701.45m)	378.688m (-422.023m to 1,501.99m)	980.643m (93.0843m to 2,489.64m)	475.63m (56.2859m to 1,116.88m)	2,607.53m (622.748m to 5,982.04m)	925.067m (259.583m to 2,088.69m)	1,338.61m (438.197m to 2,896.74m)	576.923m (195.948m to 1,255.38m)
Net monetary benefit in billion (valuing QALYs at $100,000)	30-64	1,602.06m (-14,127.7m to 11,023.6m)	-352.919m (-7,414.36m to 3,886.65m)	-8,477.07m (-26,108.9m to 1,193.28m)	-4,292.45m (-11,787.3m to 46.2403m)	-10,891.4m (-26,365.9m to -2,193.51m)	-5,222.1m (-12,334m to -1,277.75m)	-14,034.1m (-31,275.1m to -4,424.7m)	-6,319.33m (-13,896.4m to -2,096.89m)
	65-84	5,184.66m (1,676.25m to 7,924.1m)	1,060.19m (-165.786m to 2,179.1m)	192.247m (-1,355.54m to 1,256.49m)	-105.502m (-796.707m to 440.451m)	-1,499.81m (-4,824.28m to 468.796m)	-656.01m (-1,788.01m to 20.1005m)	-1,128.53m (-2,663.46m to -219.382m)	-512.555m (-1,180.4m to -125.654m)
Incremental cost-effectiveness ratio (2017 US Dollars per QALY)	30-64	87,130.3 (13,097.8 to 219,531)	107,769 (20,991.8 to 269,309)	253,691 (78,552.4 to 586,556)	305,708 (98,046.3 to 710,333)	609,912 (199,065 to 1,378,620)	733,727 (249,861 to 1,688,960)	1,587,190 (577,756 to 3,534,210)	1,884,620 (677,249 to 4,287,080)
	65-84	9,817.36 (-28,549.6 to 68,509.2)	25,997.6 (-28,846.5 to 115,172)	84,676.1 (7,608.5 to 227,698)	130,719 (12,580.8 to 430,029)	238,326 (55,882.2 to 569,892)	331,745 (92,614.3 to 826,391)	625,364 (197,597 to 1,491,120)	889,151 (252,742 to 2,592,680)

Table S8. Results of one-way sensitivity analysis, varying willingness to pay (WTP) for 1 QALY, and discount rate. Net monetary benefit ($). Brackets indicate 95% uncertainty intervals.

Population	Discount rate	WTP for QALY ($)	Long term reformulation	Short term reformulation
Food system - ever	0%	50000	2,325.28m (-18,859m to 15,573.2m)	-876.314m (-8,951.42m to 4,487.91m)
Food system - ever	0%	100000	14,941.4m (-6,535.13m to 29,085.2m)	3,122.94m (-5,291.65m to 8,845.77m)
Food system - ever	0%	150000	27,504.8m (5,073.64m to 42,999.1m)	7,209.12m (-1,543.88m to 13,484.9m)
Food system - ever	3%	50000	-2,169.8m (-21,278m to 9,127.36m)	-2,416.15m (-10,723.7m to 2,488.66m)
Food system - ever	3%	100000	6,798m (-12,056.1m to 18,370.9m)	681.259m (-7,297.79m to 5,760.18m)
Food system - ever	3%	150000	15,823m (-3,649.92m to 28,016.8m)	3,751.96m (-4,327.36m to 9,228.3m)
Food system - ever	6%	50000	-4,946.99m (-22,381.1m to 4,932.64m)	-3,413.2m (-11,468.4m to 1,260.58m)
Food system - ever	6%	100000	1,631.67m (-15,298m to 11,788.6m)	-1,008.65m (-8,858.47m to 3,822.42m)
Food system - ever	6%	150000	8,096.04m (-9,229.68m to 18,549.9m)	1,384.36m (-6,492.76m to 6,375.11m)
Food system - ever	9%	50000	-6,433.56m (-22,261.4m to 2,400.71m)	-4,143.22m (-11,905.9m to 337.231m)
Food system - ever	9%	100000	-1,690.97m (-17,102m to 7,362.65m)	-2,195.26m (-10,015.8m to 2,407.3m)
Food system - ever	9%	150000	3,051.42m (-12,492.5m to 12,500.7m)	-273.827m (-8,052.68m to 4,398.33m)
Food system - current	0%	50000	-11,706.8m (-32,253.7m to 273.587m)	-5,235.7m (-13,363m to -480.947m)
Food system - current	0%	100000	-6,908.63m (-28,343.4m to 5,049.94m)	-3,670.8m (-11,854.7m to 1,189.76m)

Food system - current	0%	150000	-2,214.22m (-23,319.3m to 10,050.2m)	-2,132.84m (-10,448.9m to 2,863.2m)
Food system - current	3%	50000	-11,805.6m (-30,196.9m to -1,082.49m)	-5,636m (-13,526.1m to -1,004.4m)
Food system - current	3%	100000	-8,330.08m (-27,466m to 2,219.04m)	-4,360.54m (-12,470.5m to 262.379m)
Food system - current	3%	150000	-4,992.06m (-24,293.3m to 5,754.12m)	-3,124.29m (-11,292.1m to 1,599.22m)
Food system - current	6%	50000	-11,573.6m (-28,302m to -1,871.38m)	-5,847.58m (-13,703.4m to -1,338.37m)
Food system - current	6%	100000	-8,996.09m (-26,122.5m to 538.321m)	-4,865.31m (-12,765m to -305.281m)
Food system - current	6%	150000	-6,484.34m (-24,102.5m to 2,995.52m)	-3,863.86m (-11,824.1m to 668.419m)
Food system - current	9%	50000	-11,127.9m (-26,549.3m to -2,303.52m)	-5,969.04m (-13,810.7m to -1,508.07m)
Food system - current	9%	100000	-9,264.27m (-24,786.4m to -477.26m)	-5,162.84m (-13,054m to -709.917m)
Food system - current	9%	150000	-7,372.3m (-23,131.5m to 1,351.22m)	-4,368.16m (-12,291.9m to 82.8871m)
Processed food - ever	0%	50000	-14,660.9m (-35,451.8m to -2,806.89m)	-6,266.64m (-14,618.2m to -1,532.78m)
Processed food - ever	0%	100000	-12,353.4m (-33,441.8m to -467.908m)	-5,524.67m (-14,007.8m to -786.541m)
Processed food - ever	0%	150000	-10,045.9m (-31,410.6m to 1,812.17m)	-4,814.67m (-13,269.1m to -56.5734m)
Processed food - ever	3%	50000	-14,006.5m (-32,811.6m to -3,273.64m)	-6,436.42m (-14,672.4m to -1,816.84m)
Processed food - ever	3%	100000	-12,375m (-31,392m to -1,670.59m)	-5,868.13m (-14,199.9m to -1,225.73m)
Processed food - ever	3%	150000	-10,766.1m (-29,972.4m to 70.7536m)	-5,329.76m (-13,718.8m to -668.255m)
Processed food - ever	6%	50000	-13,208.1m (-30,322.2m to -3,489.52m)	-6,545.58m (-14,635.4m to -2,001.88m)

Processed food - ever	6%	100000	-12,049.5m (-29,301.5m to -2,372.02m)	-6,089.21m (-14,262.2m to -1,507.41m)
Processed food - ever	6%	150000	-10,886.3m (-28,280.8m to -1,182m)	-5,645.46m (-13,889m to -1,087.74m)
Processed food - ever	9%	50000	-12,404.8m (-28,035.8m to -3,708.44m)	-6,567.5m (-14,536.7m to -2,110.33m)
Processed food - ever	9%	100000	-11,570.6m (-27,288.9m to -2,779.8m)	-6,218.55m (-14,236.5m to -1,718.48m)
Processed food - ever	9%	150000	-10,680.6m (-26,542m to -1,930.32m)	-5,853.02m (-13,936.4m to -1,362.09m)
Processed food - current	0%	50000	-17,203.2m (-37,930.2m to -5,481.03m)	-7,084.52m (-15,454.6m to -2,397.4m)
Processed food - current	0%	100000	-16,371.9m (-37,138.1m to -4,608.89m)	-6,822.93m (-15,200.1m to -2,076.6m)
Processed food - current	0%	150000	-15,572.9m (-36,346.1m to -3,829.33m)	-6,540.17m (-14,945.6m to -1,850.06m)
Processed food - current	3%	50000	-15,763.5m (-34,501.1m to -5,205.81m)	-7,051.87m (-15,275.6m to -2,449.91m)
Processed food - current	3%	100000	-15,152.5m (-33,938.6m to -4,576.3m)	-6,848.68m (-15,078.8m to -2,192.61m)
Processed food - current	3%	150000	-14,607m (-33,376.1m to -3,988.7m)	-6,625.12m (-14,882.1m to -1,967.36m)
Processed food - current	6%	50000	-14,457.2m (-31,487.8m to -4,897.38m)	-6,985.54m (-15,078.7m to -2,454.54m)
Processed food - current	6%	100000	-14,020.3m (-31,082m to -4,464.53m)	-6,825.67m (-14,923.7m to -2,262.88m)
Processed food - current	6%	150000	-13,602.4m (-30,676.3m to -4,053.96m)	-6,656.79m (-14,768.7m to -2,082.3m)
Processed food - current	9%	50000	-13,295.4m (-28,852.3m to -4,570.97m)	-6,906.27m (-14,869.6m to -2,442.62m)
Processed food - current	9%	100000	-12,965.5m (-28,554.4m to -4,244.54m)	-6,770.77m (-14,745.1m to -2,297.19m)
Processed food - current	9%	150000	-12,652m (-28,256.5m to -3,929.8m)	-6,643.83m (-14,620.7m to -2,152.56m)

REFERENCES

1. Strazzullo P, D'Elia L, Kandala N-B, Cappuccio FP. Salt intake, stroke, and cardiovascular disease: meta-analysis of prospective studies. *BMJ*. 2009;339:b4567. doi:10/fh3vbq

2. He FJ, Li J, MacGregor GA. Effect of longer-term modest salt reduction on blood pressure. In: *Cochrane Database of Systematic Reviews*. John Wiley & Sons, Ltd; 2013. http://onlinelibrary.wiley.com/doi/10.1002/14651858.CD004937.pub2/abstract. Accessed May 27, 2015.

3. Mozaffarian D, Fahimi S, Singh GM, et al. Global sodium consumption and death from cardiovascular causes. *N Engl J Med*. 2014;371(7):624-634. doi:10/f6db5p

4. World Health Organisation. *Guideline: Sodium Intake for Adults and Children*. Geneva: World Health Organisation; 2012. http://www.ncbi.nlm.nih.gov/books/NBK133309/. Accessed July 17, 2016.

5. U.S. Department of Health and Human Services, U.S. Department of Agriculture. Dietary guidelines for Americans 2015-2020. Eighth edition. December 2015. https://health.gov/dietaryguidelines/2015/resources/2015-2020_Dietary_Guidelines.pdf. Accessed May 31, 2017.

6. Ioannidis JP. Commentary: Salt and the assault of opinion on evidence. *Int J Epidemiol*. 2016;45(1):264-265. doi:10/gc5p6q

7. Mente A, O'Donnell M, Rangarajan S, et al. Associations of urinary sodium excretion with cardiovascular events in individuals with and without hypertension: a pooled analysis of data from four studies. *The Lancet*. 2016;388:465-475. doi:10/bhv7

8. O'Donnell M, Mente A, Rangarajan S, et al. Urinary sodium and potassium excretion, mortality, and cardiovascular events. *N Engl J Med*. 2014;371(7):612-623. doi:10/f249wk

9. Cogswell ME, Mugavero K, Bowman BA, Frieden TR. Dietary sodium and cardiovascular disease risk — measurement matters. *N Engl J Med*. 2016;375:580--586. doi:10/gc5p6f

10. Campbell NRC. Dissidents and dietary sodium: concerns about the commentary by O'Donnell et al. *Int J Epidemiol*. December 2016:dyw292. doi:10/gc5p5d

11. Taylor RS, Ashton KE, Moxham T, Hooper L, Ebrahim S. Reduced Dietary Salt for the Prevention of Cardiovascular Disease: A Meta-Analysis of Randomized Controlled Trials (Cochrane Review). *Am J Hypertens*. 2011;24(8):843-853. doi:10.1038/ajh.2011.115

12. Aburto NJ, Ziolkovska A, Hooper L, Elliott P, Cappuccio FP, Meerpohl JJ. Effect of lower sodium intake on health: systematic review and meta-analyses. *BMJ*. 2013;346:f1326. doi:10/f43xz8

13. Lawes CMM, Hoorn SV, Law MR, Elliott P, MacMahon S, Rodgers A. *Comparative Quantification of Health Risks. Chapter 6: High Blood Pressure*. Geneva: World Health Organisation; 2004. http://www.who.int/publications/cra/en/.

14. Williamson P. The role of the International Journal of Microsimulation. *Int J Microsimulation*. 2007;1(1):1-2.

15. Zucchelli E, Jones AM, Rice N. The evaluation of health policies through dynamic microsimulation methods. *Int J Microsimulation*. 2012;5(1):2–20.

16. Ruggles S, Genadek K, Goeken R, Grover J, Sobek M. Integrated Public Use Microdata Series: Version 7.0 [American Community Survey 2010-2014]. 2017. https://doi.org/10.18128/D010.V7.0. Accessed March 26, 2018.

17. R Core Team. *R: A Language and Environment for Statistical Computing*. R Foundation for Statistical Computing; 2014. http://www.R-project.org/.

18. Dowle M, Short T, Lianoglou S, Srinivasan A. *Data.Table: Extension of Data.Frame*.; 2015. https://github.com/Rdatatable/data.table/.

19. Revolution Analytics, Weston S. *Foreach: Foreach Looping Construct for R*.; 2014. http://CRAN.R-project.org/package=foreach.

20. Gaujoux R. *DoRNG: Generic Reproducible Parallel Backend for Foreach Loops*.; 2014. http://CRAN.R-project.org/package=doRNG.

21. L'Ecuyer P. Good Parameters and Implementations for Combined Multiple Recursive Random Number Generators. *Oper Res*. 1999;47(1):159-164. doi:10/dqx877

22. Alfons A, Kraft S, Templ M, Filzmoser P. Simulation of close-to-reality population data for household surveys with application to EU-SILC. *Stat Methods Appl*. 2011;20(3):383-407. doi:10/fs79dj

23. Kypridemos C. Modelling the effectiveness and equity of primary prevention policies in England: a stochastic dynamic microsimulation for the joint prevention of non communicable diseases. October 2016. https://elements.liverpool.ac.uk/repository.html?pub=0&com=get-file&rfurl=http%3A%2F%2Flivrepository.liverpool.ac.uk%2Frt4eprints%2Ffile%2F87606%2F201001644_Oct2016.pdf. Accessed April 11, 2017.

24. Centers for Disease Control and Prevention (CDC), National Center for Health Statistics (NCHS). National Health and Nutrition Examination Survey data. Hyattsville, MD: U.S. Department of Health and Human Services, Centers for Disease Control and Prevention. 2014 1999. https://wwwn.cdc.gov/nchs/nhanes/ContinuousNhanes/. Accessed November 15, 2016.

25. R Core Team. *R: A Language and Environment for Statistical Computing*. R Foundation for Statistical Computing; 2017. http://www.R-project.org/.

26. Meindl B, Templ M, Alfons A, Kowarik A. *SimPop: Simulation of Synthetic Populations for Survey Data Considering Auxiliary Information*.; 2017. https://CRAN.R-project.org/package=simPop.

27. Rhodes DG, Murayi T, Clemens JC, Baer DJ, Sebastian RS, Moshfegh AJ. The USDA Automated Multiple-Pass Method accurately assesses population sodium intakes. *Am J Clin Nutr*. 2013;97(5):958-964. doi:10/gc5p4t

28. Capewell S, O'Flaherty M. Rapid mortality falls after risk-factor changes in populations. *The Lancet*. 2011;378(9793):752-753. doi:10/bb4hgm

29. Capewell S, O'Flaherty M. Can dietary changes rapidly decrease cardiovascular mortality rates? *Eur Heart J*. 2011;32(10):1187-1189. doi:10/dzjxgw

30. Levin ML. The occurrence of lung cancer in man. *Acta - Unio Int Contra Cancrum*. 1953;9(3):531-541.

31. Smolina K, Wright FL, Rayner M, Goldacre MJ. Determinants of the decline in mortality from acute myocardial infarction in England between 2002 and 2010: linked national database study. *BMJ*. 2012;344:d8059. doi:10/fxxzwt

32. Young F, Capewell S, Ford ES, Critchley JA. Coronary mortality declines in the U.S. between 1980 and 2000. *Am J Prev Med*. 2010;39(3):228-234. doi:10/ckmnfd

33. Unal B, Critchley JA, Capewell S. Explaining the decline in coronary heart disease mortality in England and Wales between 1981 and 2000. *Circulation*. 2004;109(9):1101-1107. doi:10/fgtksb

34. Ford ES, Ajani UA, Croft JB, et al. Explaining the decrease in U.S. deaths from coronary disease, 1980-2000. *N Engl J Med*. 2007;356(23):2388-2398. doi:10/ftsn7g

35. Benjamin EJ, Blaha MJ, Chiuve SE, et al. Heart Disease and Stroke Statistics—2017 Update: A Report From the American Heart Association. *Circulation*. 2017;135(10):e146-e603. doi:10/cck5

36. Ford ES, Roger VL, Dunlay SM, Go AS, Rosamond WD. Challenges of Ascertaining National Trends in the Incidence of Coronary Heart Disease in the United States. *J Am Heart Assoc*. 2014;3(6):e001097. doi:10/gc5p4s

37. United States Department of Health and Human Services (US DHHS), Centers for Disease Control and Prevention (CDC), National Center for Health Statistics (NCHS). Underlying cause of death 1999–2015 on CDC WONDER online database. Data are compiled from data provided by the 57 vital statistics jurisdictions through the Vital Statistics Cooperative Program. 2016. https://wonder.cdc.gov/ucd-icd10.html. Accessed April 18, 2017.

38. Wilson PWF, D'Agostino RB, Levy D, Belanger AM, Silbershatz H, Kannel WB. Prediction of coronary heart disease using risk factor categories. *Circulation*. 1998;97(18):1837-1847. doi:10/m5c

39. Barendregt JJ, van Oortmarssen GJ, Vos T, Murray CJ. A generic model for the assessment of disease epidemiology: the computational basis of DisMod II. *Popul Health Metr*. 2003;1:4. doi:10/bgr3m7

40. Lim SS, Vos T, Flaxman AD, et al. A comparative risk assessment of burden of disease and injury attributable to 67 risk factors and risk factor clusters in 21 regions, 1990–2010: a systematic analysis for the Global Burden of Disease Study 2010. *The Lancet*. 2012;380(9859):2224-2260. doi:10/j3t

41. Boshuizen HC, Lhachimi SK, Baal PHM van, et al. The DYNAMO-HIA Model: An Efficient Implementation of a Risk Factor/Chronic Disease Markov Model for Use in Health Impact Assessment (HIA). *Demography*. 2012;49(4):1259-1283. doi:10/f4jzrb

42. Wolf P A, D'Agostino R B, Belanger A J, Kannel W B. Probability of stroke: a risk profile from the Framingham Study. *Stroke*. 1991;22(3):312-318. doi:10.1161/01.STR.22.3.312

43. D'Agostino R B, Wolf P A, Belanger A J, Kannel W B. Stroke risk profile: adjustment for antihypertensive medication. The Framingham Study. *Stroke*. 1994;25(1):40-43. doi:10.1161/01.STR.25.1.40

44. Hyndman RJ. *Demography: Forecasting Mortality, Fertility, Migration and Population Data*.; 2017. http://CRAN.R-project.org/package=demography.

45. Hyndman RJ, Shahid Ullah M. Robust forecasting of mortality and fertility rates: A functional data approach. *Comput Stat Data Anal*. 2007;51(10):4942-4956. doi:10/c4cgvx

46. Hyndman RJ, Booth H, Yasmeen F. Coherent mortality forecasting: the product-ratio method with functional time series models. *Demography*. 2013;50(1):261-283. doi:10/f4m7zw

47. Stringhini S, Carmeli C, Jokela M, et al. Socioeconomic status and the 25 × 25 risk factors as determinants of premature mortality: a multicohort study and meta-analysis of 1·7 million men and women. *The Lancet*. 2017;389(10075):1229-1237. doi:10/bzzj

48. Sullivan PW, Ghushchyan V. Preference-Based EQ-5D Index Scores for Chronic Conditions in the United States. *Med Decis Making*. 2006;26(4):410-420. doi:10/fndjhd

49. Khavjou O, Phelps D, Leib A. *Projections of Cardiovascular Disease Prevalence and Costs: 2015–2035. Technical Report*. RTI International; 2016. https://www.heart.org/idc/groups/heart-public/@wcm/@adv/documents/downloadable/ucm_491513.pdf. Accessed July 10, 2017.

50. Joo H, Dunet DO, Fang J, Wang G. Cost of informal caregiving associated with stroke among the elderly in the United States. *Neurology*. 2014;83(20):1831-1837. doi:10/f6pg4w

51. Leal J, Luengo-Fernández R, Gray A, Petersen S, Rayner M. Economic burden of cardiovascular diseases in the enlarged European Union. *Eur Heart J*. 2006;27(13):1610-1619. doi:10/dj8jrz

52. New York City Health Department. National Salt Reduction Initiative (NSRI). https://www1.nyc.gov/site/doh/health/health-topics/national-salt-reduction-initiative.page. Published 2016. Accessed July 17, 2017.

53. Food and Drug Administration (FDA), Department of Health and Human Services (DHHS). *Food and Drug Administration Justification of Estimates for Appropriations Committees. Fiscal Year 2012*. Food and Drug Administration (FDA); 2012. https://www.fda.gov/downloads/AboutFDA/ReportsManualsForms/Reports/BudgetReports/UCM243370.pdf. Accessed July 10, 2017.

54. Collins M, Mason H, O'Flaherty M, Guzman-Castillo M, Critchley J, Capewell S. An economic evaluation of salt reduction policies to reduce coronary heart disease in England: a policy modeling study. *Value Health J Int Soc Pharmacoeconomics Outcomes Res*. 2014;17(5):517-524. doi:10/f2w77b

55. Mary K. Muth, Samantha Bradley, Jenna Brophy, et al. Reformulation Cost Model. Contract No. HHSF-223-2011-10005B, Task Order 20. August 2015.

56. Food and Drug Administration (FDA). Draft guidance for industry: voluntary sodium reduction goals: target mean and upper bound concentrations for sodium in commercially processed, packaged, and prepared foods. 2016. https://www.fda.gov/downloads/Food/GuidanceRegulation/GuidanceDocumentsRegulatoryInformation/UCM503798.pdf. Accessed July 15, 2017.

57. Food and Drug Administration (FDA). FNDDS Mapping File Request 082516. https://www.regulations.gov/document?D=FDA-2014-D-0055-0410. Published 2014. Accessed July 15, 2017.

58. U.S. Department of Agriculture, Agricultural Research Service. USDA Food and Nutrient Database for Dietary Studies, 5.0. 2012. https://www.ars.usda.gov/northeast-area/beltsville-md/beltsville-human-nutrition-research-center/food-surveys-research-group/. Accessed July 15, 2017.

59. Poti JM, Dunford EK, Popkin BM. Sodium reduction in US households' packaged food and beverage purchases, 2000 to 2014. *JAMA Intern Med*. 2017;177(7):986-994. doi:10/gc5p4x

60. Koerkamp BG, Stijnen T, Weinstein MC, Hunink MGM. The combined analysis of uncertainty and patient heterogeneity in medical decision models. *Med Decis Making*. 2011;31(4):650-661. doi:10/cdf2wf

61. Briggs AH, Weinstein MC, Fenwick EAL, Karnon J, Sculpher MJ, Paltiel AD. Model parameter estimation and uncertainty: a report of the ISPOR-SMDM modeling good research practices Task Force-6. *Value Health*. 2012;15(6):835-842. doi:10/f39s8w

62. Jones AM, Lomas J, Rice N. Applying beta-type size distributions to healthcare cost regressions. *J Appl Econom*. 2014;29(4):649-670. doi:10/f5ztkb

63. Singh GM, Danaei G, Farzadfar F, et al. The age-specific quantitative effects of metabolic risk factors on cardiovascular diseases and diabetes: a pooled analysis. *PLOS ONE*. 2013;8(7):e65174. doi:10/f23vn5

64. Neumann PJ, Cohen JT, Weinstein MC. Updating cost-effectiveness — the curious resilience of the $50,000-per-QALY threshold. *N Engl J Med*. 2014;371(9):796-797. doi:10/gc5p4r

65. Micha R, Peñalvo JL, Cudhea F, Imamura F, Rehm CD, Mozaffarian D. Association between dietary factors and mortality from heart disease, stroke, and type 2 diabetes in the United States. *JAMA*. 2017;317(9):912-924. doi:10/gc5p4z
